# Linking Combustion-Derived Magnetite and Black Carbon: Insights from Magnetic Characterization of PM_2.5_ in Downwind East Asia

**DOI:** 10.1021/acs.est.4c14187

**Published:** 2025-05-22

**Authors:** Nozomu Tsuchiya, Fumikazu Ikemori, Kazuo Kawasaki, Reina Yamada, Mitsuhiko Hata, Masami Furuuchi, Yoko Iwamoto, Naoki Kaneyasu, Yasuhiro Sadanaga, Takahiro Watanabe, Takayuki Kameda, Masayo Minami, Toshio Nakamura, Atsushi Matsuki

**Affiliations:** † Graduate School of Natural Science & Technology, 12858 Kanazawa University, Kakuma-machi, Kanazawa 920-1192, Japan; ‡ Graduate School of Energy Science, 12918 Kyoto University, Yoshida-Honmachi, Sakyo-ku, Kyoto 606-8501, Japan; § Institute of Nature and Environmental Technology, Kanazawa University, Kakuma-machi, Kanazawa 920-1192, Japan; ∥ Nagoya City Institute for Environmental Science, 5-16-8 Toyoda, Minami-ku, Nagoya 457-0841, Japan; ⊥ Institute for Space−Earth Environmental Research, Nagoya University, Furo-cho, Chikusa-ku, Nagoya 464-8601, Japan; # School of Sustainable Design, 34823 University of Toyama, 3190 Gofuku, Toyama 930-8555, Japan; ¶ Graduate School of Biosphere Science, Hiroshima University, 1-7-1 Kagamiyama, Higashi-Hiroshima 739-8521, Japan; ∇ Atmospheric Environment Research Group, National Institute of Advanced Industrial Science and Technology, 1-1-1 Higashi, Tsukuba 305-8561, Japan; ○ Fukushima Institute for Research, Education and Innovation, 40-1 Yazawa-machi, Gongendo, Namie-machi, Fukushima 979-1521, Japan; ⧫ Graduate School of Engineering, 12936 Osaka Metropolitan University, 1-1 Gakuen-cho, Naka-ku, Sakai 599-8531, Japan; †† Tono Geoscience Center, 50169 Japan Atomic Energy Agency, 959-31 Jorinji, Izumi-cho, Toki 509-5102, Japan

**Keywords:** magnetite, iron oxides, black carbon, carbon isotopes, combustion emissions

## Abstract

Combustion-derived magnetite has recently attracted attention for its health risks and potential impact on atmospheric heating/cooling. This study provides new observational insights into the relationship between black carbon (BC) and magnetite at a remote site in East Asia, Japan, focusing on combustion sources, seasonal trends, and potential overestimation of BC by the light-absorbing magnetite. Magnetic measurements of PM_2.5_ samples, complemented by detailed chemical analyses, reveal similar temporal variations between BC and magnetite while demonstrating that the relative abundance of magnetite to BC varies by combustion source, driving seasonal trends. Magnetite abundance during combustion episodes was found to follow the order: coal > oil > biomass, with mass concentrations roughly estimated via magnetization to be 9–10%, 5–6%, and <2% of BC, respectively. Furthermore, magnetite was estimated to contribute up to 5% of the BC absorption coefficient, suggesting the considerable overestimation of BC depending on the source. Although regionality and source mixing should be further verified, these findings show that magnetic measurements of archived samples can offer valuable contributions to reconstructing long-term combustion trends or overestimates in conventional observations of BC.

## Graphical abstract

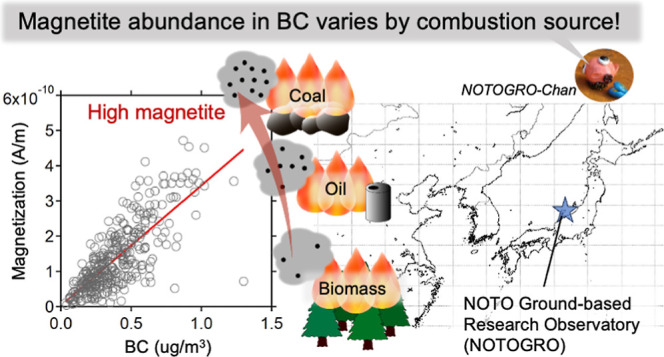

## Introduction

Iron oxide nanoparticles (FeO_*x*_), including magnetite (Fe_3_O_4_), maghemite (γ-Fe_2_O_3_), and hematite (α-Fe_2_O_3_), are increasingly recognized as significant atmospheric components.^1–3^ These aerosols originate from both anthropogenic activities, such as fossil fuel combustion, traffic, and iron/steel production, as well as natural sources like soil and desert dust.^4–10^ Among these, magnetite is particularly noteworthy as a combustion-derived component due to its toxicity, which has been linked to neurodegenerative diseases such as Alzheimer’s and Parkinson’s.^11–16^ While magnetite is often associated with coarse aerosols, especially in regions downwind of major natural desert sources,^7,10^ combustion-derived magnetite can penetrate deeper into the human body (e.g., brain and heart) via respiratory/olfactory pathways and bloodstream due to its inherently small size.^11,17,18^

From a climate perspective, magnetite is considered a potential contributor to both global warming and cooling. While black carbon (BC), a well-known combustion-derived aerosol,^19–21^ has been regarded as the primary light-absorbing aerosol component, recent studies highlight the contribution of combustion-derived magnetite to atmospheric heating due to its light absorption, similar to BC.^2,22^ In contrast, iron deposition via aeolian transport may enhance marine productivity in high-nitrate, low-chlorophyll (HNLC) regions such as the northern Pacific, thereby resulting in cooling effect.^23,24^ Although magnetite itself is water-insoluble, proton/ligand-promoted and photoreductive dissolution processes can increase the solubility of such insoluble species.^25–32^ Several observations have reported internal mixing of iron-containing nanoparticles with acids (e.g., sulfate, nitrate, and oxalate),^1,3,31–35^ suggesting a potential role for combustion-derived magnetite in stimulating marine productivity. Monitoring magnetite is therefore critical for understanding geochemical cycles and climate dynamics.

Another key consideration in this study is that the light absorption of magnetite may lead to overestimation of BC, which is conventionally measured based on aerosol light absorption. In such case, magnetite compromises the accuracy of BC monitoring and climate impact assessments due to its dual role in atmospheric heating (similar to BC) and cooling (via ocean deposition). In addition, reported variations in iron content across different combustion sources suggest that the relative abundance of magnetite to BC varies depending on the emission source,^36,37^ potentially leading to or exacerbating BC overestimations. These concerns underscore the need for separate monitoring of BC and magnetite to accurately assess the climate impacts of combustion-related aerosols.

East Asia, a global hotspot for atmospheric aerosol emissions,^38^ offers a unique opportunity to study the relationships between BC, magnetite, and their combustion sources. The monsoon-driven outflow from the East Asian continent facilitates long-range aerosol transport from diverse combustion sources, leading to pronounced seasonality in carbonaceous components in downwind regions like Japan.^39–44^ Previous study using single-particle soot photometer (SP2) measurements identified abundant FeO_*x*_, predominantly magnetite, associated with BC in the East Asian outflows, with an estimated contribution to atmospheric heating of at least 4–7% of BC.^2^ Additionally, the emission flux of anthropogenic FeO_*x*_ from China has been estimated at 0.21–0.49 FeTg/yr.^33^ However, these studies using SP2 relied on short-term observations and did not quantify magnetite within FeO_*x*_, highlighting the need for long-term evaluations specifically focusing on magnetite to better understand its sources and climate impacts. Furthermore, the extent to which BC measurements have been overestimated due to magnetite mixing remains unclear.

This study addresses these gaps by investigating fine aerosols (PM_2.5_) collected at a remote site in Japan. Using magnetic measurements capable of distinguishing magnetite from hematite,^7,8,10,45^ in conjunction with conventional BC observations via multiangle absorption photometer (MAAP) and detailed chemical analyses of carbon isotopes, biomass burning tracers, and metal elements, we aim to elucidate the long-term behavior of combustion-derived magnetite. Specifically, this study examines the relative abundance of magnetite to BC, its source-dependent seasonal trends, and its contribution to BC overestimation, providing new insights into the environmental and climatic roles of magnetite.

## Materials and Methods

### Aerosol Sampling and Observations

Aerosol sampling and observation were conducted from June 2014 to October 2015 at an atmospheric monitoring supersite (NOTO Ground-based Research Observatory, NOTOGRO, 37.45° N, 137.36° E) of Kanazawa University, Japan, located at the tip of the Noto Peninsula (Figure S1). It is a remote coastal site facing the Asian continent and is isolated from major cities and industrial activities by the surrounding sea. Such a location is considered ideal for monitoring background aerosol properties in East Asia without the significant influence of local anthropogenic sources^46,47^ and for sensitively detecting slight changes in atmospheric composition associated with continental outflow. Daily and weekly PM_2.5_ samples were collected by a model 5012 MAAP (Thermo Fisher Scientific Inc.) and a high-volume air sampler (HV) (AH-600F, SIBATA Scientific Technology Ltd.). Details of aerosol sampling are found in the Supporting Information (Section S1.1). Daily and weekly samples underwent magnetic and detailed chemical analyses, respectively. Two parameters relating to combustion emissions were observed at the same location: (1) the mass concentration of BC by MAAP based on light absorption/scattering at a wavelength of 670 nm and (2) the volume concentration of CO by a nondispersive infrared photometer (model 48i; Thermo Fisher Scientific Inc.).

### Isothermal Remanent Magnetization Measurements of Daily Samples

Daily PM_2.5_ samples were cut out spot by spot using ceramic scissors and placed in nonmagnetic plastic cubes (7 cm^3^).^10^ IRM measurements were conducted using a pulse magnetizer (PM9, Magnetic Measurements) with a direct-current (DC) magnetic field, followed by measurements with a cryogenic magnetometer (750, 2G Enterprises). Two magnetization steps were applied to each sample: a 1.2 T DC field to obtain saturation IRM (IRM_1.2_), and second, a −0.3 T DC field to acquire the IRM in the opposite direction (IRM_–0.3_). These measurements reflect the contributions of total magnetic particles (IRM_1.2_) and soft (low-coercivity) magnetic particles, such as magnetite (IRM_–0.3_). The *S*-ratio (*S*_–0.3_)^48,49^ was calculated to characterize the magnetic mineralogy of the samples as follows

1 $$S‐ratio\left(S_{-0.3}\right)=\left[1+\left({IRM}_{-0.3}{/IRM}_{1.2}\right)\right]/2$$

Here, IRM intensities are positive values (>0), and the *S*-ratio ranges from 0 to 1. A value closer to 1 indicates a higher contribution of soft magnetic particles (e.g., magnetite), as their IRM saturates at fields <0.3 T, unlike hard (high-coercivity) magnetic particles (e.g., hematite), which do not saturate at 0.3 T. Detailed results of the *S*-ratio, along with additional low-temperature magnetic analysis, are provided in Section S2, confirming that soft magnetic component is predominantly represented by magnetite. In this study, the soft IRM intensity was calculated using the following formula as an index of magnetite concentration

2 $$IRM={IRM}_{1.2}\times S‐ratio$$

IRM intensities are reported in units of A/m, representing magnetization (Am^2^) normalized by the sampled air volume (m^3^).

### Chemical Analyses of Weekly Samples

To determine the total carbon (TC) content, including organic carbon (OC) and elemental carbon (EC), of weekly samples prior to isotopic analysis, Interagency Monitoring of Protected Visual Environments (IMPROVE) Thermal-Optical Reflectance (TOR) analysis was conducted using a Lab OC-EC Aerosol Analyzer (Sunset Laboratory Inc.).^50^

Carbon isotopic analysis required the postsampling treatments for graphitization (Section S1.3).^40^ The prepared graphite sample was loaded in an aluminum sample holder, and the carbon isotopic composition (^14^C/^12^C and ^13^C/^12^C) of TC was analyzed using the accelerator mass spectrometry (AMS) ^14^C system (High Voltage Engineering Europe, model 4130-AMS).^51^ Based on the carbon isotopic composition, the concentration of modern carbon (^14^C) was expressed by the following equations

3 C14concentration(pMC)=(14C/12C)sample,corr/(14C/12C)AD1950×100=(14C/12C)sample,corr/[(0.7459)×(14C/12C)HOxII,corr]×100

The unit of ^14^C concentration is shown in terms of percent modern carbon (pMC). The constant 0.7459 is the correction factor to convert ^14^C/^12^C of HOxII into the standard ^14^C concentration used in carbon chronological dating. The expression “corr” denotes the δ^13^C within each sample measured by the AMS normalized to −25‰ to correct for the isotopic fractionation.

The fraction of the CO_2_ sample was separated during the postsampling process, and ^13^C concentration was analyzed for the selection of samples using isotope ratio mass spectrometry (IRMS, Finnigan MAT252, Thermo Scientific). Results are expressed in terms of δ^13^C (‰) relative to standard Vienna Pee Dee Belemnite (VPDB), which is defined as

δC13(‰)=[(C13/C12)]sample/[(C13/C12)VPDB−1]×1000 4

In addition, two biomass burning tracers, levoglucosan and mannosan–characteristic of organic compounds in the smoke^52–54^ –were extracted from weekly samples and analyzed using GC–MS (GC: Agilent 7890A, MS: Agilent 5975C) based on the method described by previous reports.^55,56^

Metal elements, including vanadium (V) and lead (Pb), were analyzed using an inductively coupled plasma mass spectrometry (ICP-MS) instrument (X, Thermo Elemental) following sample dissolution (detailed in Section S1.4).

## Results and Discussion

### Temporal/Seasonal Variation of Aerosol Magnetization

In this study, the soft IRM intensity, derived from eq 2, was used to interpret variations in IRM as proxies for magnetite (Section S2). Notably, aerosol IRM at the study site can be strongly influenced by the influx of Asian dust.^10^ Because the focus here is on combustion-related magnetite, samples with anomalously high IRM, likely attributable to Asian dust, were excluded. Their exclusions were based on two criteria: extremely high IRM values (>95th percentile for the entire study period) and abrupt IRM increases relative to BC concentrations (>14 day moving average +1σ in terms of the IRM/BC). After the screening, the BC concentrations ranged from 0.03 to 1.30 μg/m^3^ with an average of 0.39 μg/m^3^, and the IRM intensities ranged from 0.05 to 4.72 × 10^–10^ A/m, with an average of 1.35 × 10^–10^ A/m, over the study period (*n* = 348). Among the excluded samples, the highest IRM intensity recorded was 2.88 × 10^–9^ A/m, higher than 10 times the average, observed on March 29, 2015, during a large-scale Asian dust event.^10^

Temporal variations of BC mass concentration and IRM intensity on a daily scale exhibited a striking similarity throughout most of the study period (Figure 1a). In Japan, the transboundary air pollution from the Asian continent is known to increase PM concentrations, particularly during winter and spring seasons.^41,44,57–60^ This phenomenon likely explains the observed winter-spring increases in BC and IRM (Figure S4). Furthermore, the BC versus IRM plot (Figure 1b) and their strong correlation (Pearson’s correlation coefficient of 0.77, *p* < 0.0001) suggest a shared origin for magnetite and BC, primarily from combustion emissions associated with the continental outflow. However, some deviations between BC and IRM were observed, such as relatively high BC concentrations compared to IRM in late July and mid-October 2014. These discrepancies, along with scatter in the BC-IRM relationship, indicate that BC and magnetite are not always emitted at uniform rates. The variations likely depend on their respective emission sources, assuming that BC and magnetite have similar atmospheric lifetimes. In this study, three combustion modes—biomass, oil, and coal-related emissions—were examined as potential contributors to the observed dispersion in the relative abundance of magnetite to BC.

**Figure 1:**
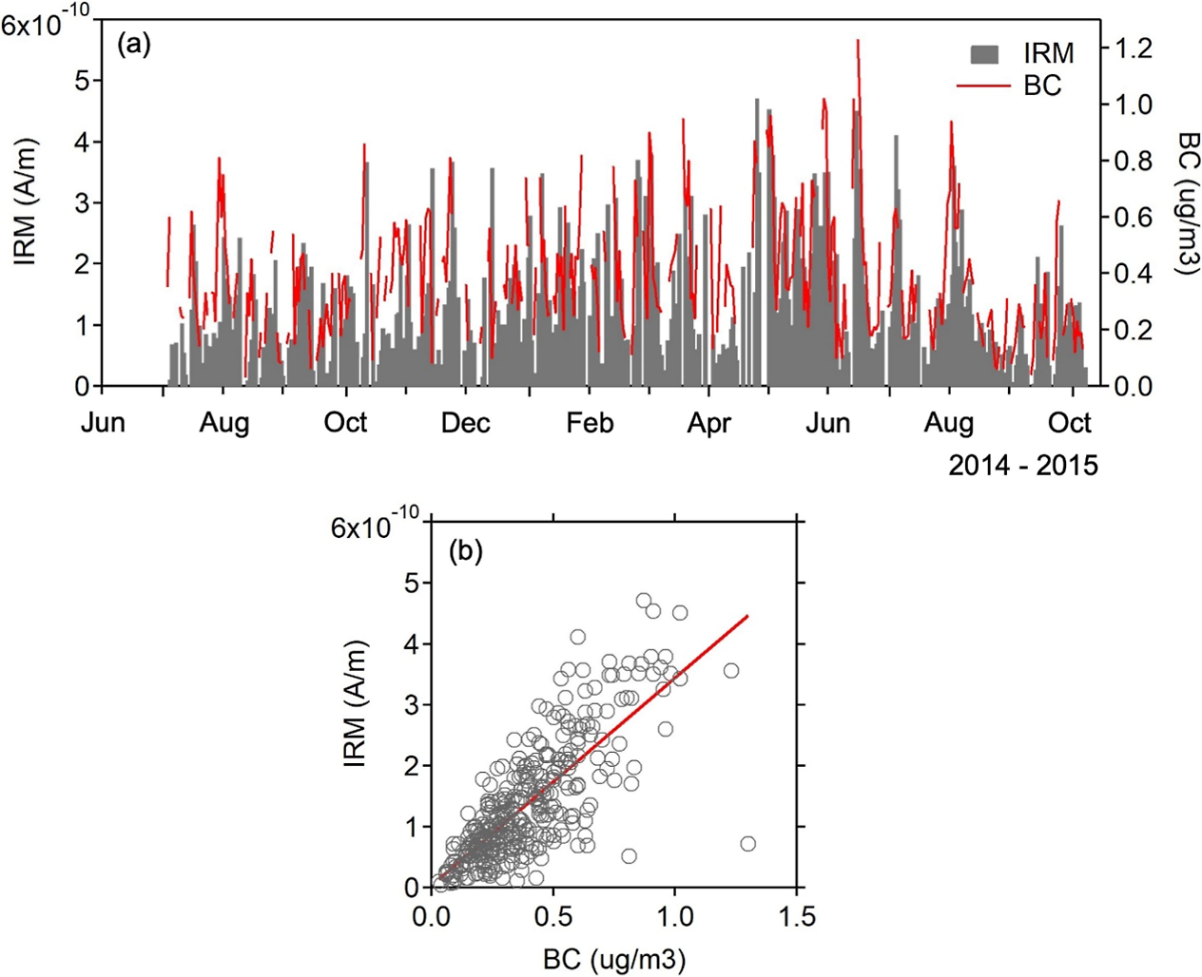
(a) Temporal variations of BC mass concentration and IRM intensity obtained from the daily aerosol samples and (b) their correlation (*n* = 348, *R* ^2^ = 0.60, *p* < 0.0001).

### Identification of Specific Combustion Sources

To identify specific combustion events, detailed chemical analyses were conducted on weekly HV samples, including measurements of modern carbon (^14^C), stable carbon (δ^13^C), biomass burning tracers (levoglucosan, mannosan, and their ratio L/M), and metal elements (V and Pb). The ^14^C concentrations ranged from 57.4 to 89.6 pMC (Figure 2), showing seasonal variation (further details in Section S3). Background levels were higher in summer (∼70 pMC) compared to winter (∼60 pMC), a trend also reflected in the OC variation (Figure S5). This seasonal pattern suggests that a high photochemical production of secondary organic aerosol (SOA) in summer is a contributing factor.^61,62^ Notable ^14^C increases were observed in late July and October 2014 (Figure 2a), indicating large-scale biomass burning events. These events were further corroborated by variations in levoglucosan, mannosan, and δ^13^C values.

**Figure 2:**
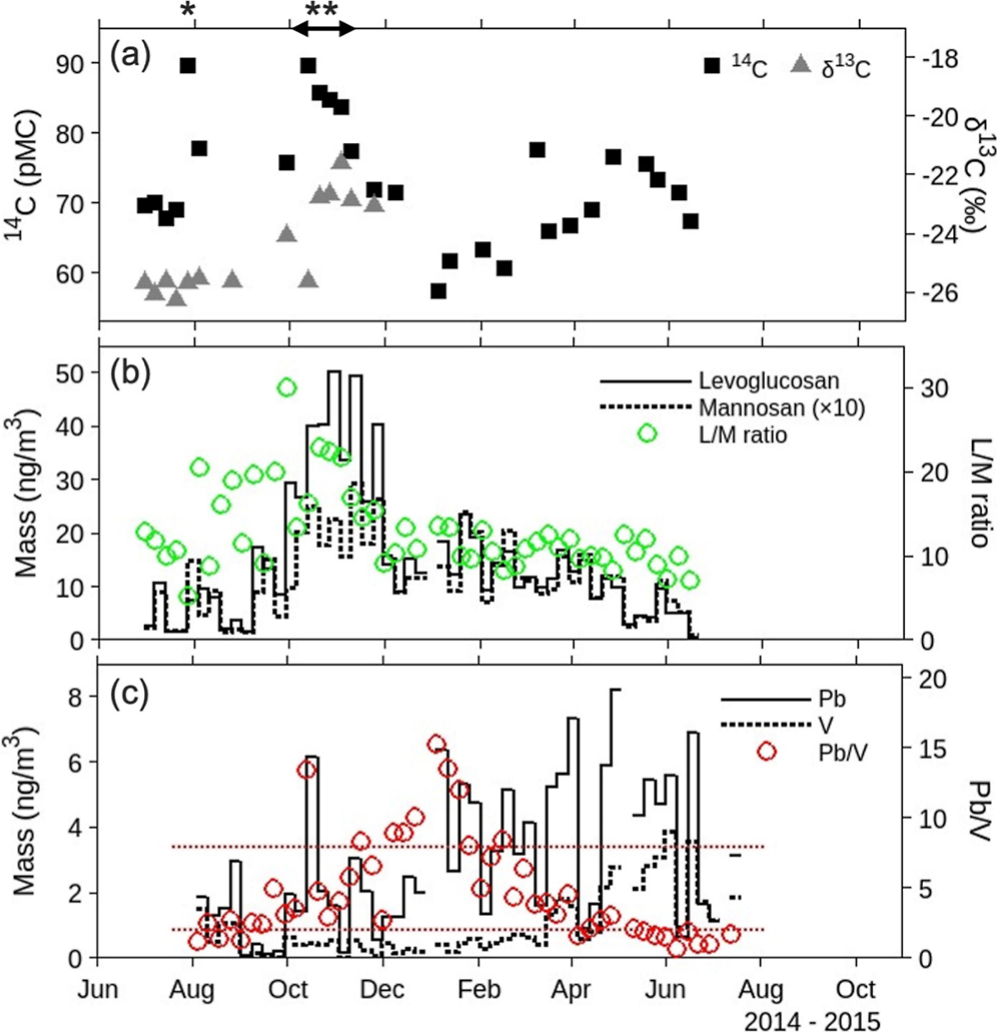
Temporal variations of (a) ^14^C and δ^13^C, (b) levoglucosan and mannosan, and (c) Pb and V, obtained from weekly samples. Asterisk denotes periods influenced by Siberian forest fire (*) and open burning in Northeastern China (**), identified in Section S4. Red dashed lines in (c) represent Pb/V of 2 and 8, corresponding to lower and upper 20th percentile.

Details of biomass source identification are provided in Section S4. Briefly, the ^14^C spike in late July 2014 was attributed to Siberian forest fire. This conclusion was supported by the L/M ratio, which reached its lowest value of 5.1 (Figure 2b), and backward trajectory analysis using HYSPLIT combined with fire-spot locations detected by the MODIS satellite sensor (Figure S6). This finding highlights the significant influence of continental biomass emissions on carbonaceous aerosols even in summer, when domestic emissions typically dominate (Figure S7). The ^14^C spike and subsequent plateau in October 2014 were attributed to open burning of C_4_ crop residues, likely maize straw, in Northeastern China. This conclusion was based on biomass burning tracers, the δ^13^C values, and backward trajectories linked to fire-spot locations (Figure S6). Such agricultural burning practices can exert substantial and prolonged impacts on downwind regions, comparable to those caused by large-scale Siberian forest fires through continental outflow.

In contrast to the ^14^C peaks, relatively low ^14^C concentrations were observed during winter. This trend can be attributed to shifts in primary aerosol emission sources, as well as reduced SOA production. In November, when residential heating from coal combustion begins in Northeastern China,^63–66^ δ^13^C values remained elevated (>−25‰) despite the decline in ^14^C concentrations, indicative of reduced open biomass burning activity. The sustained high δ^13^C values can be explained by coal combustion (δ^13^C: −25‰ to −23‰), which produces higher δ^13^C compared to oil combustion (δ^13^C: −30‰ to −24‰). This suggests a seasonal shift toward coal as a dominant fuel source in winter. The lower δ^13^C values observed in summer (<−25‰) align with this interpretation.

Seasonal trends were further corroborated by the concentrations of Pb and V used as tracers to distinguish between coal and oil combustion, respectively.^67–69^ The Pb/V ratio was notably higher in winter compared to summer (Figure 3c), indicating an increased contribution of coal combustion to fossil fuel emissions in winter, while oil combustion was more prominent in summer. Backward trajectories suggest that these emissions originate from both continental and domestic sources (Figure S7). These findings align with previous studies in Japan, which reported a greater contribution of coal combustion to PM in winter.^41,44,57^ This seasonal pattern reflects the higher reliance on coal for energy production in China compared to Japan, compounded by additional emissions from residential coal-fired heating in China during the colder months.

**Figure 3:**
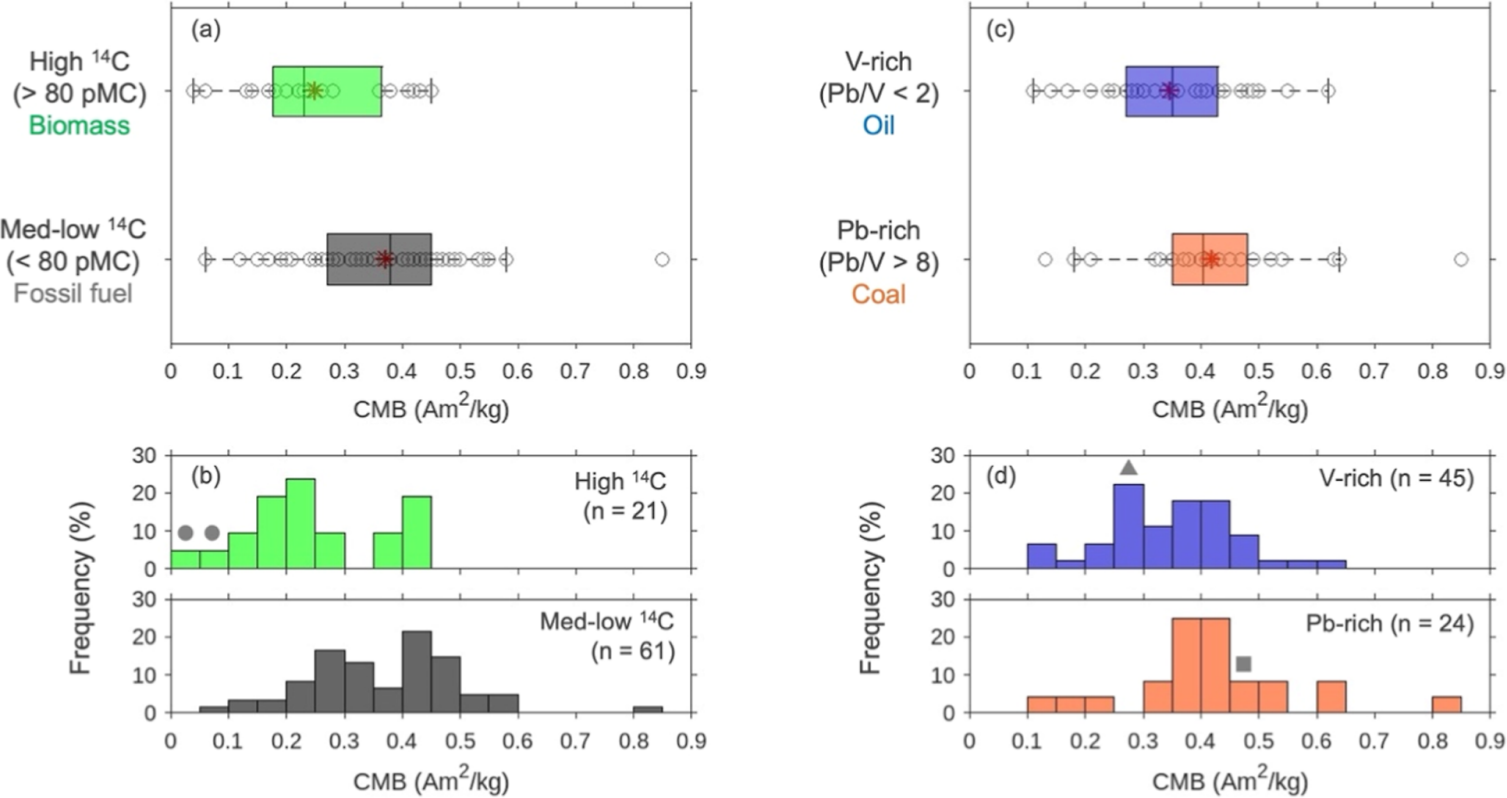
CMB distribution in the different sample groups based on ^14^C concentration and Pb/V ratio obtained from weekly HV samples. (a–d) separate samples into the high (>80 pMC)/medium-low ^14^C (<80 pMC) concentration groups, and the V-rich (Pb/V < 2)/Pb-rich (Pb/V > 8) groups with significance (*p* values of 0.0004 and 0.0393 respectively, by Wilcoxon rank-sum test). Red asterisk represents the average for each group. Gray markers denote that CMBs of the typical combustion events (circle: biomass, triangle: oil, square: coal) are found in the corresponding bins.

### Correspondence between Combustion Source and Magnetite

This section evaluates the contribution of combustion modes (biomass, oil, and coal) to the relative abundance of magnetite to BC (i.e., IRM/BC expressed in Am^2^/kg). This metric was calculated by dividing IRM intensity (A/m) by BC concentration (μg/m^3^), effectively representing the slope of the BC vs IRM plot from the origin to each data point (Figure 1b). To ensure an unbiased analysis of combustion influences on the IRM/BC value, CO concentration was used as an independent proxy for combustion. Specifically, samples with ΔCO (the increase from the CO baseline, defined as the 14 day moving fifth percentile) exceeding 20 ppb were selected (*n* = 208).^70^ The IRM/BC values of these samples were defined as the combustion magnetization-BC ratio (CMB).

To determine the influence of biomass burning to CMB, samples were categorized based on their ^14^C concentrations, and their CMB values were compared. The high ^14^C group (>80 pMC), which included notable biomass burning events, exhibited significantly lower CMB values compared to the medium-low ^14^C group (<80 pMC) (Figure 3a), with a statistically significant difference (*p* = 0.0004, Wilcoxon rank-sum test). It is important to note that the high ^14^C classification was on a weekly basis and may include individual days with lower ^14^C levels (this is the same for the following discussion of fossil fuels). Three distinct CMB peaks were identified in terms of frequency: low (0.2–0.25 Am^2^/kg), medium (0.25–0.35 Am^2^/kg), and high (0.4–0.5 Am^2^/kg). Notably, the low CMB peak was characteristic of the high ^14^C group (Figure 3b), suggesting that magnetite is less abundant in forest fire and open burning emissions, which are characterized by low CMB values. Specifically, extremely low CMB values of 0.06 and 0.04 Am^2^/kg (highlighted by circles in Figure 3b) were observed on July 30 and October 29, 2014, respectively. Recall that there were periods with increased BC while IRM remained low in July and October (Figure 1a). These dates correspond to the Siberian forest fire^54^ and the day reported also by Uranishi et al. (2019) as having high contribution of open burning in Northeastern China to PM_2.5_ in Japan.^18^ This finding suggests that pure biomass burning emissions can drive the CMB value down to ∼0.04 Am^2^/kg. The more frequent low CMB values around 0.2 Am^2^/kg likely reflect a mixing of biomass and fossil fuel emissions.

The contribution of fossil fuel combustion to CMB was evaluated by categorizing samples based on their Pb/V ratio, a tracer for distinguishing between coal and oil combustion. Samples with Pb/V < 2 (lower 20th percentile) were classified as V-rich (oil-dominant), while those with Pb/V > 8 (upper 20th percentile) were categorized as Pb-rich (coal-dominant). The V-rich group exhibited significantly lower CMB values compared to the Pb-rich group (*p* = 0.0393, Wilcoxon rank-sum test). In terms of frequency distribution, the V-rich group displayed medium (0.25–0.3 Am^2^/kg) and high (0.35–0.45 Am^2^/kg) CMB peaks, while the Pb-rich group was characterized exclusively by the high CMB peak (0.35–0.45 Am^2^/kg). It is expected that the medium CMB peak in the V-rich group is attributed to oil combustion, while the high CMB peak in the Pb-rich group is indicative of coal combustion, and this means the enrichment of magnetite especially in coal combustion emissions. This observation aligns with a report of abundant submicron magnetic particles in coal combustion emissions.^71^ Notably, the lowest and highest Pb/V periods (representing typical oil and coal combustion events, respectively) corresponded to CMB values of 0.25 and 0.48 Am^2^/kg (triangle and square markers in Figure 3d), highlighting the characteristic enrichment of magnetite in coal-derived emissions. On this basis, the frequent CMB values in 0.2–0.25 Am^2^/kg and 0.35–0.45 Am^2^/kg can be interpreted as indicative of the mixing of biomass and fossil fuel combustion and oil and coal combustion, respectively. Besides, CMB values exceeding 0.5 Am^2^/kg were observed in some cases, with the highest value (0.85 Am^2^/kg) occurring in December 2014. This sample had a moderate ^14^C concentration (71.4 pMC) and a relatively high Pb/V ratio (8.9), suggesting a coal combustion influence. The week also included days with extremely high IRM, of which samples were screened out due to suspected dust influence. This suggests that small-scale dust influxes may occasionally evade screening and contribute to elevated CMB values. Pure coal combustion could also yield CMB values >0.5 Am^2^/kg, particularly when weekly chemical analyses smooth out and fail to capture daily variations in coal contributions. While this study site is situated to minimize the influence of traffic or industrial iron/steel production—significant contributors to urban atmospheric iron in China,^6,8,35^ —the occasional contribution of such emissions in the continental outflow, leading to elevated CMB values >0.5 Am^2^/kg, cannot be entirely excluded.

These findings suggest that magnetite is mixed with BC in combustion emissions to varying degrees, with the CMB hierarchy reflecting source contributions (coal > oil > biomass) in East Asia. This characteristic does not conflict with the reported iron content in combustion emissions.^36,37^ However, it is also essential to consider the influence of combustion conditions—particularly temperature—since the chemical composition of emitted particles can vary even when the fuel type remains constant. To assess this effect, the ratio char-/soot-EC, derived from OC-EC analysis, was compared with CMB values (Figure S8). Soot-EC is typically produced under high-temperature combustion, while char-EC is more representative of lower-temperature conditions.^72,73^ Additionally, oil combustion is generally associated with a higher soot-EC fraction, whereas char-EC is characteristically emitted from coal combustion—especially from low-temperature sources like residential stoves—and from biomass burning.^74,75^ Most samples, including those identified as influenced by oil or coal combustion (as shown in Figure 3), exhibited low char-/soot-EC ratios (∼1 or lower). In contrast, samples attributed to biomass burning consistently showed higher char-/soot-EC ratios (>1). Magnetite aerosol formation is favored at elevated temperatures exceeding ∼1000 °C, where iron-bearing minerals (e.g., pyrite) are converted and supersaturated iron vapors are formed.^37,71,76^ Therefore, biomass burning is unlikely to be a significant source of magnetite, owing to its typically lower combustion temperatures and lower iron emissions. This aligns with our observations of low CMB values (<0.1 Am^2^/kg) and high char-/soot-EC ratios during large-scale burning events, such as forest fire and open-field burning. Conversely, high CMB values (>0.25 Am^2^/kg) were mostly associated with low char-/soot-EC ratios (<1), suggesting that magnetite formation is enhanced under high-temperature combustion conditions. These results support the interpretation that the oil and coal combustion events contributing to elevated CMB in this study are primarily associated with high-temperature sources—such as industrial boilers, power plants, and vehicle exhaust—rather than lower-temperature residential combustion sources.^75^

Based on the observed relationships among samples associated with representative combustion events, the following CMB values are proposed as endmembers for combustion source identification: <0.1 Am^2^/kg for biomass burning, 0.25–0.3 Am^2^/kg for oil combustion, and 0.45–0.5 Am^2^/kg or more for coal combustion (higher temperature). CMB values between these ranges are interpreted as mixed-source contributions. On this basis, the CMB seasonality at the study location (Figure 4) was interpreted as follows. In spring and winter, the CMB peak was at ∼0.4 Am^2^/kg, suggesting that the dominant combustion emission was represented by a mixing of oil and coal. In summer, CMB peaks were found at ∼0.3 Am^2^/kg and higher than 0.4 Am^2^/kg, with the former likely reflecting that the dominant combustion source in summer is oil, but with a separate contribution from coal, i.e., a relatively unmixed source on certain days during this season. In autumn, the CMB showed a broad distribution with frequent CMB values <0.2 Am^2^/kg, suggesting a stronger biomass contribution compared to other seasons. A significant difference was found between summer and winter by the Kruskal–Wallis test (*p* = 0.026). These trends are consistent with the chemical analyses of weekly samples, which revealed a difference in fuel types between summer and winter and a prolonged contribution from open burning in Northeastern China during autumn (Figure 2).

**Figure 4:**
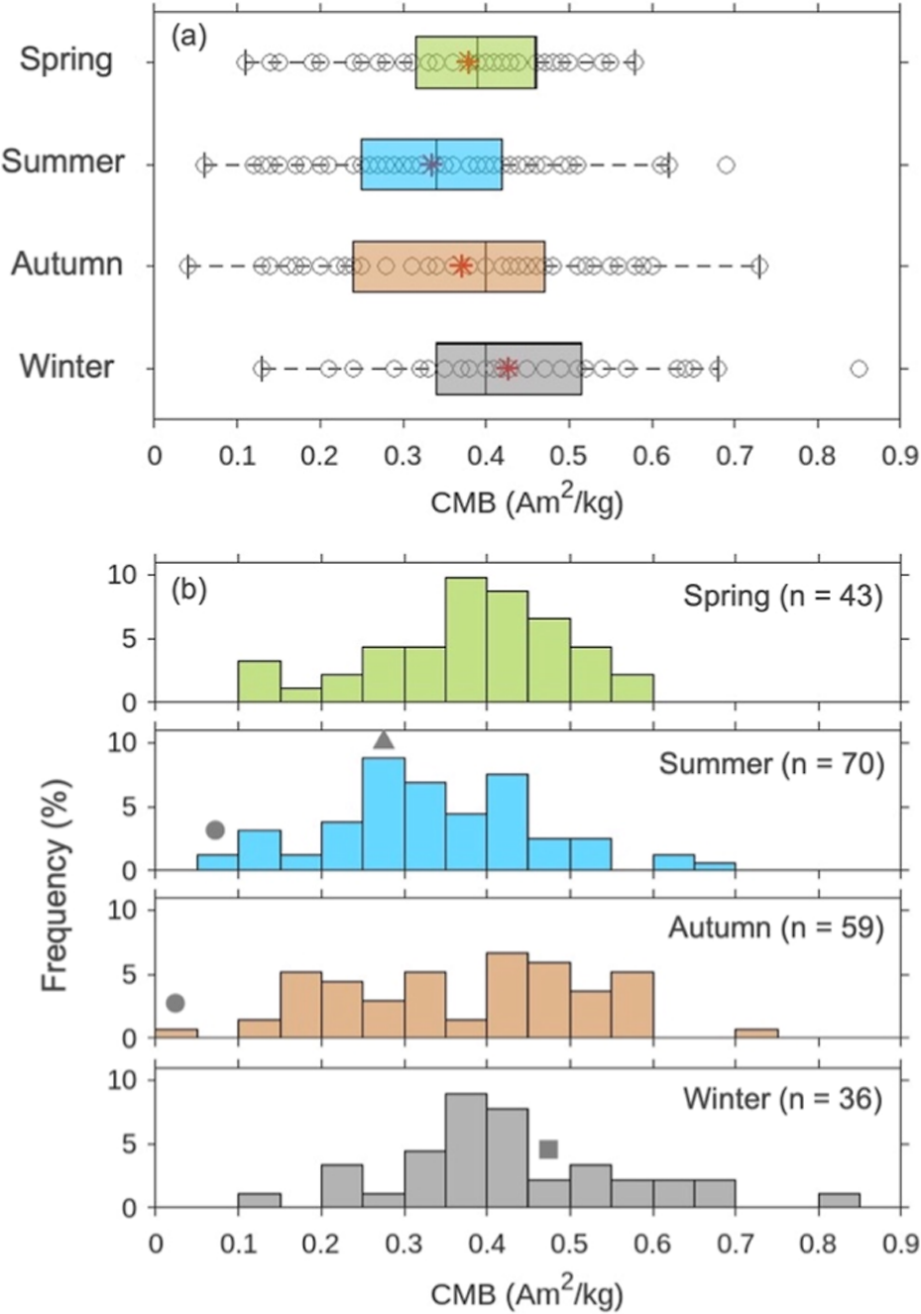
Seasonal variation of CMB (combustion magnetization-BC ratio) by (a) box plot and (b) frequency distribution (*n* = 208 in total). Red marker (*) represents seasonal average: 0.38 Am^2^/kg in Spring (Mar–May), 0.33 Am^2^/kg in summer (Jun–Aug), 0.37 Am^2^/kg in autumn (Sep–Nov), and 0.43 Am^2^/kg in winter (Dec–Feb). Gray markers denote the corresponding bins where typical combustion events (circle: biomass, triangle: oil, square: coal) were found, as in Figure 3.

Thus, the observed CMB values can serve as indicators of magnetite abundance to BC and/or the dominant combustion source. The daily IRM measurement, which utilize a rapid and nondestructive technique, effectively captured source influences, aligning with insights from more detailed weekly chemical analyses and demonstrating its applicability. While the results of this study are case-specific and do not comprehensively cover magnetite sources or define strict CMB values for individual sources, the fundamental CMB hierarchy (coal > oil > biomass) is expected to be applicable elsewhere. It is also noteworthy that even in a downwind remote location with suspected complex source mixing, magnetite abundance exhibited clear daily and seasonal variations. Further investigations that account for proximity to emission sources and more precise differentiation of combustion processes (e.g., contribution from low temperature coal combustion) are needed to enhance the understanding of magnetite behavior.

### Rough Estimate of Magnetite and FeO_*x*_ Concentrations and Contribution to BC

The volume-magnetization of aerosol samples (measured in A/m) can be roughly recalculated to the mass concentrations of magnetite and FeO_*x*_ (sum of magnetite and hematite) by applying the individual mass-magnetization intensity of magnetite or hematite (reported in Am^2^/kg). In addition to the soft IRM, which is represented by magnetite and mainly used for discussion, the hard IRM, which is assumed to reflect hematite mass and calculated by subtracting the soft IRM from IRM at 1.2 T (refer to eq 2), was used for the estimation. The values of mass-magnetization intensity were assumed to be ∼5.0 Am^2^/kg and 0.064 Am^2^/kg for magnetite and hematite, respectively (hematite: an average of the reported hard IRM_1–0.3T_ values of two pure hematite samples).^45,77,78^ The estimated mass concentration of magnetite for combustion-affected samples (those for which the CMB was calculated) was found to be 34.1 ± 20.0 ng/m^3^, which is lower than the reported range of 75.5 ± 33.2 ng/m^3^ for Beijing, China, obtained from sequential magnetic extraction of PM_2.5_ and mass quantification.^6^ Given that Beijing is a mega-city located upwind of Japan, the range of estimated magnetite concentrations here is considered reasonable. Based on this, the classification of combustion modes (biomass, oil, and coal) through the CMB of <0.1, 0.25–0.3, and 0.45–0.5 Am^2^/kg can be directly linked to magnetite content as <2%, 5–6%, and 9–10% of BC by mass. The FeO_*x*_ mass concentration was estimated at 121.0 ± 92.3 ng/m^3^ (up to ∼400 ng/m^3^) based on the sum of magnetite and hematite. The aircraft measurements using SP2 over the Yellow and East China Seas in February–March 2013 reported FeO_*x*_ mass concentration of 100–400 ng/m^3^ (30–50% of BC) in the continental outflow below 2 km in altitude.^2^ A ground-based SP2 measurement at Cape Hedo, Okinawa (Japan) in March 2016, also reported FeO_*x*_ levels up to ∼350 ng/m^3^ (∼30% of BC).^33^ These reported values support our estimate, and it is suggested that hematite can have a non-negligible contribution to FeO_*x*_ mass concentration in SP2 measurements. In terms of relative concentration to BC, the high proportion up to 50% FeO_*x*_/BC in our estimate does not conflict with the SP2-based reports (Figure S9). Although uncertainties in the IRM (e.g., potential mixing with metallic iron)^45^ and SP2 (e.g., limited detectable size range)^2,33^ measurements should be acknowledged, the observed agreement in FeO_*x*_ concentration ranges between these different techniques underscores the potential of magnetic measurements as a complementary or alternative tool to SP2. Sequential magnetic extraction followed by mass quantification^6^ could further aid in verifying or integrating results from both observations.

Additionally, this study examines the possibility that dark-colored magnetite cause an overestimation of BC, which is traditionally measured by aerosol light absorption. To investigate this, we evaluated the potential bias in BC estimation in terms of absorption coefficient. BC mass concentration was calculated using the absorption coefficient measured at a wavelength of 670 nm and a constant mass absorption cross-section (MAC) of 6.6 m^2^/g. Magnetite mass was estimated above, and its absorption contribution was approximated assuming a MAC of 2 m^2^/g for particles with a mass equivalent diameter (*D*_m_) of ∼100–200 nm.^2,79^ Based on these assumptions, magnetite was estimated to account for 0.3%, 2%, and 3% of the BC absorption coefficient for typical biomass, oil, and coal combustion, respectively, with the highest contribution reaching up to 5%. Although direct comparisons are limited by methodological differences, these estimates are comparable to those reported by Moteki et al. (2017), who attributed 4–7% of shortwave BC absorption in the continental outflows to FeO_*x*_, based on size-resolved (170 < *D*_m_ < 2100 nm) mass measurements using SP2.^2^ Our findings further emphasize the particularly low contribution of magnetite during biomass burning events.

Since MAC is highly dependent on particle size distribution, the mode diameter plays a critical role in estimating the absorption coefficient of magnetite. The rationale for selecting MAC around 100–200 nm is based on the reported magnetite particle size distributions that exhibit peaks around 50–150 nm. These size distributions have been observed in magnetite particles originating from the road traffic, coal fly ash, and PM_2.5_ samples collected near Beijing following magnetic extraction.^6^ Additionally, it should be noted that the presence of coatings on magnetite particles can enhance their optical properties.^1,3,34^ Previous studies suggest that such coatings can increase the MAC by up to ∼1.5–2 times compared to uncoated (bare) magnetite.^2^ Therefore, further investigation into the size distribution and mixing state of magnetite, particularly in the Asian continental outflow, is essential for improving the accuracy of light absorption estimates.

## Environmental Implications

This study successfully demonstrated the seasonality of magnetite from different combustion sources in the East Asian continental outflow, showing the applicability of aerosol magnetism as a useful indicator of combustion-derived magnetite. The magnetization relative to BC (i.e., CMB), obtained from daily aerosol samples, exhibited significant variation depending on the combustion source: coal > oil > biomass. Specifically, CMB values were found to be 0.45–0.5 Am^2^/kg for coal, 0.25–0.3 Am^2^/kg for oil, and <0.1 Am^2^/kg for biomass burning. The seasonal variations of CMB were attributed to the characteristic contributions of coal (winter-spring), oil (summer), and biomass (autumn), as confirmed by detailed chemical analyses, including carbon isotopes of TC. These findings suggest that magnetite-related health risks, atmospheric heating, and oceanic iron deposition (indirect cooling) may vary with combustion sources and seasons. Regarding marine productivity, magnetite from fossil fuel combustion may become more soluble through interactions with coemitted acid species (e.g., sulfate, nitrate, and oxalate).^25–32^ Expanding CMB data set and comparing it with iron solubility might therefore provide deeper insights into iron geochemical cycle. Note that dust influx or other anthropogenic activities (e.g., traffic and iron/steel making) can also increase CMB, and the specific values for each combustion source may vary by study location or combustion conditions (e.g., variations in raw materials and temperature).

The mass concentration of combustion-derived magnetite was roughly estimated to be 34.1 ± 20.0 ng/m^3^, which is in an agreement with values reported for Beijing (China) but much lower than FeO_*x*_ concentrations observed in the East Asian downwind region, as measured by SP2. After correcting for hematite mass (based on hard magnetization), the FeO_*x*_ mass was estimated at 121.0 ± 92.3 ng/m^3^ (up to ∼400 ng/m^3^), with a relative concentration to BC typically less than 50%. This estimate aligns with SP2-based reports, supporting the credibility of mass estimates derived from aerosol magnetization. Although applying magnetic measurements to real-time atmospheric monitoring, such as SP2, is currently limited by instrumentation challenges, the nondestructive and instantaneous nature of this method makes it particularly well-suited for studies involving large volumes of archived samples. Magnetic investigations on archived samples can recover valuable past or long-term observational data, providing a complementary approach to detailed chemical analyses for source identification or magnetite (FeO_*x*_) measurements by SP2.

Moreover, the findings of this study are expected to improve climate effect estimations related to magnetite or FeO_*x*_. A previous study, which assumed all SP2-measured FeO_*x*_ was magnetite and that the emission rates of magnetite and BC were constant across sources, investigated the radiative effects and iron deposition to the ocean caused by anthropogenic magnetite.^22^ In contrast, this study estimates magnetite to be 9–10% of BC by mass for coal combustion, 5–6% for oil combustion, and <2% for biomass burning. This suggests that the SP2-based FeO_*x*_ concentrations may overestimate the contribution of magnetite, as they do not sufficiently account for the mixing with hematite. The overestimation of magnetite could lead to an inaccurate estimate of its climate impacts, especially in potential cases of which hematite is a significant component of FeO_*x*_. This study also evaluated the potential overestimation of BC owing to the light-absorbing properties of magnetite. Magnetite originating from coal and oil combustion was estimated to contribute more significantly—up to 5%—to the BC absorption coefficient, whereas its contribution during biomass burning events was comparatively minor.

Overall, further studies using aerosol magnetization, which provides complementary information to traditional chemical analyses and observational instruments, are anticipated to yield a deeper understanding of the climate and health impacts associated with human activities.

## Supplementary Material



## Notes

Additional methods (Section S1); magnetic properties (Section S2); the contribution of modern carbon (Section S3); identification of source biomass of the remarkable ^14^C increase (Section S4); Figures S1–S9 and Table S1 (PDF)
