# Supplementary material for: Linking Combustion-Derived Magnetite and Black Carbon: Insights from Magnetic Characterization of PM2.5 in Downwind East Asia
Source: Environ Sci Technol. 2025 May 22;59(21):10400–10. doi: 10.1021/acs.est.4c14187 (PMC12138966; doi:10.1021/acs.est.4c14187)
Supplement: Supplementary file 1 [file es4c14187_si_001.pdf]

Supporting Information

Linking combustion-derived magnetite and black carbon: Insights from magnetic characterization of $PM_{2.5}$ in downwind East Asia

*Nozomu Tsuchiya[1,2], Fumikazu Ikemori[3,4,5], Kazuo Kawasaki[6], Reina Yamada[1], Mitsuhiko Hata[1], Masami Furuuchi[1], Yoko Iwamoto[7], Naoki Kaneyasu[8,9], Yasuhiro Sadanaga[10], Takahiro Watanabe[11], Takayuki Kameda[2], Masayo Minami[5], Toshio Nakamura[5], and Atsushi Matsuki[3]**

[1]Graduate School of Natural Science & Technology, Kanazawa University, Kakuma-machi, Kanazawa 920-1192, Japan

[2]Graduate School of Energy Science, Kyoto University, Yoshida-Honmachi, Sakyo-ku, Kyoto 606-8501, Japan

[3]Institute of Nature and Environmental Technology, Kanazawa University, Kakuma-machi, Kanazawa 920-1192, Japan

[4]Nagoya City Institute for Environmental Science, 5-16-8 Toyoda, Minami-ku, Nagoya 457-0841, Japan

[5]Institute for Space–Earth Environmental Research, Nagoya University, Furo-cho, Chikusa-ku, Nagoya 464-8601, Japan

[6]School of Sustainable Design, University of Toyama, 3190 Gofuku, Toyama, 930-8555, Japan

[7]Graduate School of Biosphere Science, Hiroshima University, 1-7-1 Kagamiyama, Higashi-Hiroshima 739-8521, Japan

[8]Atmospheric Environment Research Group, National Institute of Advanced Industrial Science and Technology, 1-1-1 Higashi, Tsukuba 305-8561, Japan

[9]Fukushima Institute for Research, Education and Innovation, 40-1 Yazawa-machi, Gongendo, Namie-machi, Fukushima 979-1521, Japan

[10]Graduate School of Engineering, Osaka Metropolitan University, 1-1 Gakuen-cho, Naka-ku, Sakai 599-8531, Japan

[11]Tono Geoscience Center, Japan Atomic Energy Agency, 959-31 Jorinji, Izumi-cho, Toki 509-5102, Japan

Corresponding author: Atsushi Matsuki (matsuki@staff.kanazawa-u.ac.jp)

Contents

Summary: 28 pages, 9 figures, 1 table

1. Supplementary method

1.1. Aerosol sampling

1.2. Low-temperature magnetic analysis of weekly samples

1.3. Sample preparation for radiocarbon (^{14}C) analysis

1.4. Inorganic (metal) analysis

1.5. Trajectory analysis

2. Magnetic properties

3. The contribution of modern carbon contained in Noto aerosols ($PM_{2.5}$)

4. Identification of source biomass

4.1. The remarkable ^{14}C increase in July 2014

4.2. The remarkable ^{14}C increase in October 2014

5. Supplementary Figures

6. Supplementary Table

7. References

1. Supplementary method

1.1. Aerosol sampling

Weekly $PM_{2.5}$ samples were collected from June 2014 to March 2016. A high-volume air sampler (HV) coupled with a $PM_{2.5}$ impactor (AH-600F, SIBATA Scientific Technology Ltd.) was installed on the roof of a three-story building, approximately 13 m above ground level. The quartz fiber filter (2500QAT-UP, Pall Corporation) was covered with aluminum foil and preheated at 450 ºC for 2 h in an electric oven prior to sampling to remove organics from the filter background. After one week of continuous sampling at a flow rate of 700 L/min, the recovered filters were stored under −20 ºC to avoid contamination and loss of volatiles until further extraction and analysis. Note that the results of the detailed carbonaceous analyses presented in this study are based solely on the analysis of the weekly fine mode ($PM_{2.5}$) fraction because our focus was on fine combustion-derived aerosols.

Daily $PM_{2.5}$ samples were made automatically using a Model 5012 MAAP (Thermo Fisher Scientific Inc.), which measures the mass concentration of BC based on light absorbing/scattering at a wavelength of 670 nm. The sampling period was from July 2014 to October 2015. The sample air was introduced through a PM_{10} cut-off inlet installed at the same height as the HV and a $PM_{2.5}$ cut-off cyclone, and therefore only the $PM_{2.5}$ fractions were collected on a glass fiber tape filter (GF10, Thermo Fisher Scientific Inc.) at a flow rate of 16.7 L/min.

1.2. Low-temperature magnetic analysis of weekly samples

Low-temperature magnetic analysis was performed on the selected weekly HV samples (to obtain enough magnetization intensity) using Magnetic Property Measurement System (MPMS, Quantum Design) for identifying the dominant magnetic mineralogy. Samples were cut out by ceramic scissors and placed in a gelatin capsule prior to the measurement. Magnetization of samples were monitored during zero-field cooling to 5 K, magnetizing by a 1 T DC field, and zero-field heating to room temperature.

1.3. Sample preparation for radiocarbon (^{14}C) analysis

The details of sample preparation for radiocarbon (^{14}C) analysis are described here. Total carbon (TC) was calculated as the sum of OC and EC for each sample, and the sample size for isotopic analyses was determined based on the TC content. As mentioned in the main text, graphite samples were purified by the following steps: 1) sample isolation, 2) sample combustion, 3) CO_2 purification, and 4) graphitization. The post-

sample treatment procedure (first step) follows Ikemori et al. (2015),[1] but is slightly modified to cope with trace amounts of carbon in the samples. In brief, sample aliquots were obtained from punching holes on the aerosol laden quartz fiber filter and stuffed into a pre-heated quartz tube (9 mm in diameter). After adding pieces of Cu, CuO (500 mg), and Ag fibers (five threads of 0.1 mm thickness) together into the filter filled tube, the open end of the tube was connected to a vacuum line. The Cu, CuO, and Ag additives act as a reducing agent to convert NO_2 into N_2, oxidizer, and sulfur trap, respectively. When the vacuum level (7.0×10^{-3} torr) is reached, the open end of the quartz glass tube was sealed and isolated from the vacuum line.

The sample combustion (second step) was made by carefully controlling the heating temperatures stepwise. The tubes were placed in an electric furnace which was gradually heated up to 600 ºC over a duration of 3 h. After keeping the temperature constant at 600 ºC for 2 h, the furnace temperature was increased again to 900 ºC in 2 h and maintained at this temperature for 3 h. This gradual and stepwise heating was employed to facilitate the removal of sulfur oxides by Ag, which otherwise would deteriorate the success rate of graphitization of CO_2 in the following process. This is particularly important for processing fine mode ambient aerosol particles as they often consist large quantities of sulfates.

The CO_2 was purified (third step) by releasing the sample gas into a vacuum line but with a series of cold traps. The temperature differences of the coolants, such as liquid nitrogen (−196 ºC), ethanol (cooled to −90 ºC), n-pentane (−129.8 ºC), enables extraction of only the CO_2 by leaving water vapor, sulfur oxide, and other gases in the cold traps. Finally, graphite was formed (forth step) by the reaction of the purified CO_2 and H_2 gas with Fe catalyst using the small-mass graphitization system at 620 °C for 4 h.[2]

1.4. Inorganic (metal) analysis

Inorganic (metal) analysis was performed on weekly samples using inductively coupled plasma mass spectrometry (ICP-MS). Each sample was cut to a specific size from each filter using ceramic scissors and subjected to acid dissolution. The dissolution process involved a 24-h treatment with a mixture of HNO_3 (3 mL) and 48% HF (3 mL), followed by a 24-h treatment with 36% HCl (10 mL). The dissolved samples were diluted with 0.6% HNO_3 and analyzed using ICP-MS instrument (X, Thermo Elemental).

1.5. Trajectory analysis

In order to estimate the origin of the aerosols sampled on each filter, five-days

backward trajectories (start time: 0000 LST and 1200 LST on each day for the specific periods including biomass burning events (Figure S6), 0000LST on each day for the seasonal trend (Figure S7)) at the height of 500 m above mean sea level were calculated by using the hybrid single-particle Lagrangian integrated trajectory (HYSPLIT4) model (https://www.ready.noaa.gov/HYSPLIT.php).[3,4] Satellite based MODIS active fire product provided by NASA were also referred to (https://www.earthdata.nasa.gov/learn/find-data/near-real-time/firms/active-fire-data) as an indicator of any major biomass burning activity in the potential source regions.

2. Magnetic properties

Temporal and seasonal variations of S-ratio in daily samples are presented in Figure S2. Consistent with findings from our previous study on coarse aerosols,[5] high S-ratio values (average of 0.97) indicate a large contribution of soft (magnetite-like) magnetic particles to IRM.[6,7] The temperature-dependent remanence of the selected weekly samples exhibited a Verwey transition at approximately 120 K,[8] confirming that the dominant soft magnetic mineralogy in $PM_{2.5}$ is magnetite (Figure S3).

To focus on combustion-derived magnetite and minimize potential confusion with hematite, this study analyzed variations in the soft IRM, reflecting magnetite content (obtained by Eq. (2) in the main text). A slight decrease in the S-ratio (i.e., an increased contribution of hard magnetic particles, such as hematite) was observed in autumn (Figure S2). While this phenomenon lacks a definitive explanation, the seasonality of combustion sources discussed in SI Section 4.2 and the main text suggests that autumn is characterized by the extensive open burning in Northeastern China. As noted in the main text, biomass burning results in lower magnetite abundance relative to BC. This relative decrease may also explain the observed increase in hematite-like magnetic particles, contributing to the lower S-ratio.

Besides, the abrupt decrease at ~120 K (Figure S3) appears to be suppressed compared to the non-stoichiometric magnetite, which is attributed to partial oxidization of the magnetite surface or minor maghemite mixing.[6,9–11] This partial oxidation is not expected to affect the discussion due to the similarity in IRM intensities between magnetite and maghemite.[12] In addition, it is noted that this study cannot completely rule out the possibility of the presence of metallic iron (Fe^0), which can contribute to soft IRM.[13]

3. The contribution of modern carbon contained in Noto aerosols ($PM_{2.5}$)

The radiocarbon analytical-based ^{14}C data of fine HV aerosol samples consists of those of 27 samples in which > 90% carbon was successfully recovered as graphite during sample processing (Table S1). Despite the remoteness of the sampling site from major industrial and urban activities, significant ranges of ^{14}C (57.4–89.6 pMC) were observed throughout the year, indicating that the contribution of carbonaceous matter originating from modern biological activity and/or biomass burning is relatively large and it accounted for an annual average of 73.0 pMC. Compared to the other reported ^{14}C values from areas relevant to East Asia, for example 33−48 pMC in Beijing,[14] 24−34 pMC (Cape Hedo, Okinawa) and 18−43 pMC (Fukue island),[15] 31−54 pMC in Tokyo,[16] and 28−68 pMC in Nagoya,[1] our values (57−90 pMC) lie towards the upper end, highlighting a smaller contribution from fossil sources. The comparison with Nagoya may be particularly relevant because the city lies almost in the same longitudinal zone (35.15˚ N, 136.97˚ E) as our remote measurement site but it is one of the major cities along the Pacific coast (opposite side) of the main island of Japan. In this context, our results may be more representative of the 'background' ^{14}C concentrations in the central part of Japan, as Nagoya may be subject to the impact of large scale industries, transport, and agricultural activities within the city and its outskirts.

4. Identification of source biomass

As mentioned in the main text, ^{14}C by itself cannot actually tell if the modern carbon originates from biomass burning (e.g., forest fire, open burning of agricultural waste, and litter incinerations) or purely from natural biomass activities (e.g., plant debris or SOA from BVOCs), and levoglucosan and mannosan were analyzed and referred to narrow down the biomass source corresponding to the ^{14}C spikes (in late July and October 2014). Levoglucosan and mannosan are monosaccharide anhydrides that are specifically formed by the pyrolysis of cellulose and hemicellulose at high burning temperatures (> 300 ˚C) and therefore, these organic compounds are often used as distinct biomass burning tracer.[17,18]

If there were strong biomass burning activities along the air mass passage, noticeable increase in the organic tracers should be seen. Samples collected in autumn (October and November 2015) showed the highest levoglucosan concentrations (> 30 ng/m^3) (Figure 2b in main text). It is noteworthy that this autumn levoglucosan peak overlaps with the prolonged period with the highest contributions from modern carbon represented by 89.6 pMC in October 2014. On the other hand, levoglucosan signal was not as obvious in the case of ^{14}C spike in late July (i.e., July 23–30). Instead, this sample in late July showed noticeable increase in mannosan (an isomer of levoglucosan) concentration (1.5 ng/m^3).

These apparent and coincident increases in the modern carbon and the biomass burning tracers indicate that the maximum ^{14}C episodes observed in late July and in October were strongly influenced by biomass burning activities in upwind regions rather than the contribution from natural biological activities alone.

4.1. The remarkable ^{14}C increase in July 2014

For the late July peak of ^{14}C and mannosan, backward trajectories suggest that the air mass may had passed over the vast regions from East Siberian taiga (or boreal forest) to the north-east of Lake Baikal before passing north-east China to arrive at the sampling location (Figure S6a). The fire spots detected by the MODIS satellite sensor were concentrated in east Siberia and not as evident in north-eastern China. Jung et al. (2016) also reported an elevated concentration of levoglucosan in Daejeon, South Korea, associated with the long-range transport of smoke from the Siberian forest in late July 2014.[19] It is worth noting that levoglucosan was not particularly high during this period but a noticeable increase in mannosan was observed instead (Figure 2b). It has been reported that low levoglucosan to mannosan (L/M) ratios of 2.6–5.0 can be interpreted as an indicator of burning soft wood such as conifers.[20] The lowest L/M ratio of 5.1 was recorded for this particular sample (July 23–30), almost approaching the characteristic range of soft wood smoke. The East Siberian taiga is literally covered by coniferous forests. It may be worth pointing out that such perennial trees may potentially concentrate high ^{14}C in their wood through their growth during the era of multiple atomic bomb experiments[21] which may be partly responsible for the maximum ^{14}C value observed in this study. The ^{14}C peak, backward trajectories, fire spots, and biomass burning tracers indicate that a plume from Siberian forest fire had reached our sampling site during this particular period. This case well illustrates that a sporadic transboundary transport of continental air mass can actually take place even in summer period although it is generally believed to be blocked by the prevailing Pacific high pressure system.

4.2. The remarkable ^{14}C increase in October 2014

The coincidence of prolonged periods with ^{14}C plateau and high concentrations of levoglucosan and mannosan indicate the influence of a large scale open fire on samples collected in autumn (October 2014), however, at time scales longer than the sporadic Siberian forest fire episode. Unlike the Siberian event period, L/M ratio in October typically showed higher values (> 15). This rules out Siberian (coniferous) forest fire as the major contributor. To further narrow down the type of plants that were involved in the fire, the stable carbon isotopic ratio $\delta^{13}C$ was referred to within the subset of samples

collected around the high ^{14}C (> 80 pMC) episodes, namely, the ^{14}C spike in late July and the plateau in October 2014.

The identification of source biomass using $\delta^{13}C$ is based on the assumption that biogenic/biomass burning aerosols retain the characteristic stable carbon isotopic ratios which depend on the characteristic photosynthetic pathway of the source plant. For example, C_3 plants (including many terrestrial plants and crops such as rice, wheat, potatoes, and soy) take in ^{13}C slowly and show low $\delta^{13}C$ values in the range −30 to −25‰. C_4 plants, which typically include tropical and subtropical grasses, such as, maize and sugarcane, show smaller kinetic isotope effects, and thus, have average ratios of −15 to −10‰, which are much closer to the ratio found in the atmospheric CO_2.[22,23]

In this study, the variation of $\delta^{13}C$ values among the selection of samples from July and October 2014 were obtained (Figure 2a). During summer, the ratio was always found to be around −26‰, which is fairly close to the ratios found among C_3 plants. In particular, this includes the sample taken during the Siberian forest fire episode (July 23–30). C_4 plants do not usually grow in cool climates and more primitive trees such as conifers belong to the C_3 group. This is not surprising and does not conflict with the conclusion that this sample in late July was strongly affected by the Siberian forest fire. On the other hand, most of the samples during autumn showed values higher than −23‰. This is higher than what is expected from many C_3 plants, thus, indicating the presence of endmembers having much higher $\delta^{13}C$ ratios.

Cao et al. (2011) reported increased $\delta^{13}C$ ratio in $PM_{2.5}$ TC by about 2‰ from −26.5‰ in summer (June/July) to −24.4‰ in winter (January) in northern Chinese cities.[24] This seasonal increase, however, was mainly attributed to the increased use of coal for residential heating during the season (November to March). According to Cao et al. (2011), a formal residential heating season in North China starts in November.[24] Therefore, collective coal burning would not have started during the period in concern (October) and potential contribution from coal (fossil fuel) combustion cannot explain the dominance of modern carbon and high biomass burning tracers observed. Besides, the $\delta^{13}C$ expected from coal combustion (−25 to −23‰) and vehicular emissions (−30 to −24‰) are too low to be the predominant endmember in concern.[25]

One possible source of modern biomass having high $\delta^{13}C$ ratios may be marine phytoplankton, which typically shows −22 to −19‰,[26] similar to the values found in the carbonaceous aerosols in October. Kim et al. (2000) reported seasonal variation of pigment concentrations in different areas within the Sea of Japan and spotted a spring

peak in March to April and an autumn peak which starts in September and peaks in November.[27] Although the pigment concentration at the surface water and organic mass fraction within associated sea spray aerosols may not always be directly linked,[28] increased marine primary productivity around the area in autumn could affect the isotopic ratio of organic aerosols. However, in order for the marine aerosols to explain the observed $\delta^{13}C$ ratios near −22‰, they must dominate the carbonaceous components because the observed ratios are approaching their characteristic values (−22 to −19‰). Since relatively high concentrations of BC were observed and the samples in autumn showed persistent and strong biomass burning signals, the predominance of marine sources is ruled out. The remaining possibility, therefore, is biomass burning involving terrestrial plants enriched in ^{13}C, namely, C_4 plants.

The air-mass trajectories arriving at the sampling site in October and the fire spots detected by MODIS satellite in the corresponding periods are plotted in Figure S6b and c. There is a region with highly clustered fire spots in north-eastern China and air-mass had likely passed directly over those regions at relatively low altitudes. The coincidence of the trajectories and the dense fire spot areas is rather striking. This is most certainly where the large-scale, systematic biomass burning had been taking place which caused the high ^{14}C and strong biomass burning signals. Additionally, the observation of high $\delta^{13}C$ values in autumn indicated that the large modern carbon (non-fossil) fraction observed in the corresponding period was associated with a large-scale post-harvest burn involving C_4 plants, namely, maize straw, in the north-eastern parts of China. There are a number of reports that strongly support this view.

Wheat and maize straws form the major agricultural residues in China and field burning of such biomass is still a common practice.[29] Although such a practice has been banned since the late 90’s due to deteriorating regional air quality, major changes in the energy structure turned down the demands of the residues as an attractive energy source and facilitated field burning. Such biomass burning is expected to peak with crop harvests. Huang et al. (2012) constructed a multi-annual, emission inventory of crop burning in different parts of China based on MODIS fire products.[30] They demonstrated a strong seasonality in the agricultural fire counts in north-eastern China (which coincides with the region in concern), with peaks in spring (March, April, and May) and in autumn (October) as well. Interestingly, the inter-annual analysis between 2003–2010 revealed that the second peak in October was hardly visible in the early years, but gradually became prominent in the later years,[30] which suggests that the second peak of post-harvest burn in October in this region is a rather new and growing practice which emerged merely

over the course of the last decade or so.

Sun et al. (2016) estimated the CO_2 emissions related to open fires of major crop residues, namely, rice, wheat, and maize, in China from 1996 to 2013.[31] Their estimates suggested the emissions especially from burning maize straw have shown a sharp increase since around 2004. According to their estimate, CO_2 emissions from burning of rice, wheat, and maize straw in 2013 were 3038, 3747, and 8728 ($\times 10^4$ t), respectively, and the rate of increase relative to 2004 was 1.8 for maize (much faster than 1.3–1.4 for rice and wheat). Furthermore, this rapidly increasing trend of maize straw burning is particularly pronounced in the north-eastern prefectures, such as Heilongjiang, Jiliun, and Liaoning, which are the largest maize producing areas in the country (http://www.circleofblue.org/china-grains.html). This is due to incentives given by the local governments which motivated farmers to switch over to maize instead of producing soybeans and rice. This rapid change is said to have damaged both the grain structure as well as the local air quality in the north-eastern regions.

5. Supplementary Figures

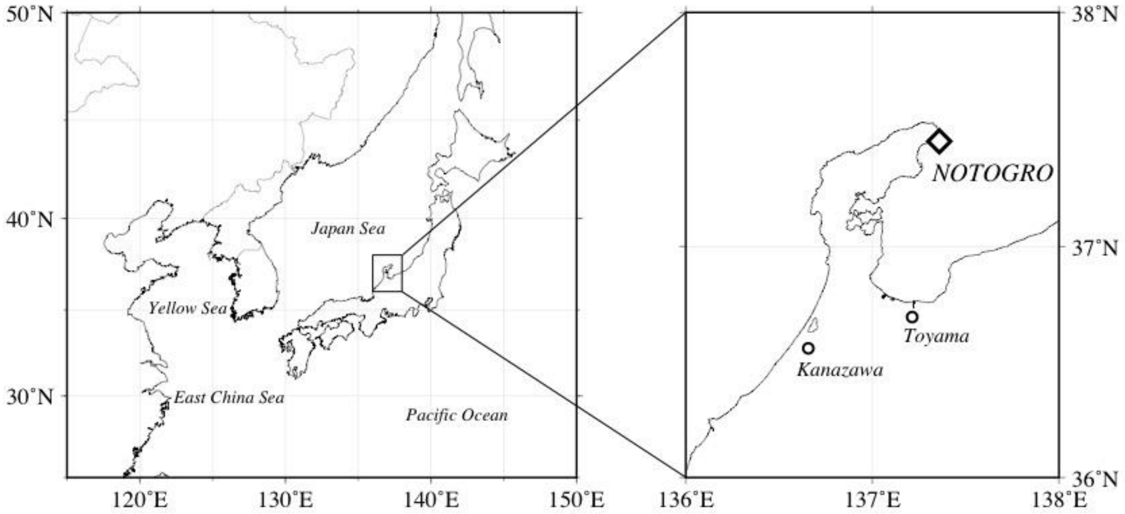

Figure S1. Geographical location of NOTOGRO station (37.45° N, 137.36° E, same location as in Tsuchiya et al., 2023).[5]

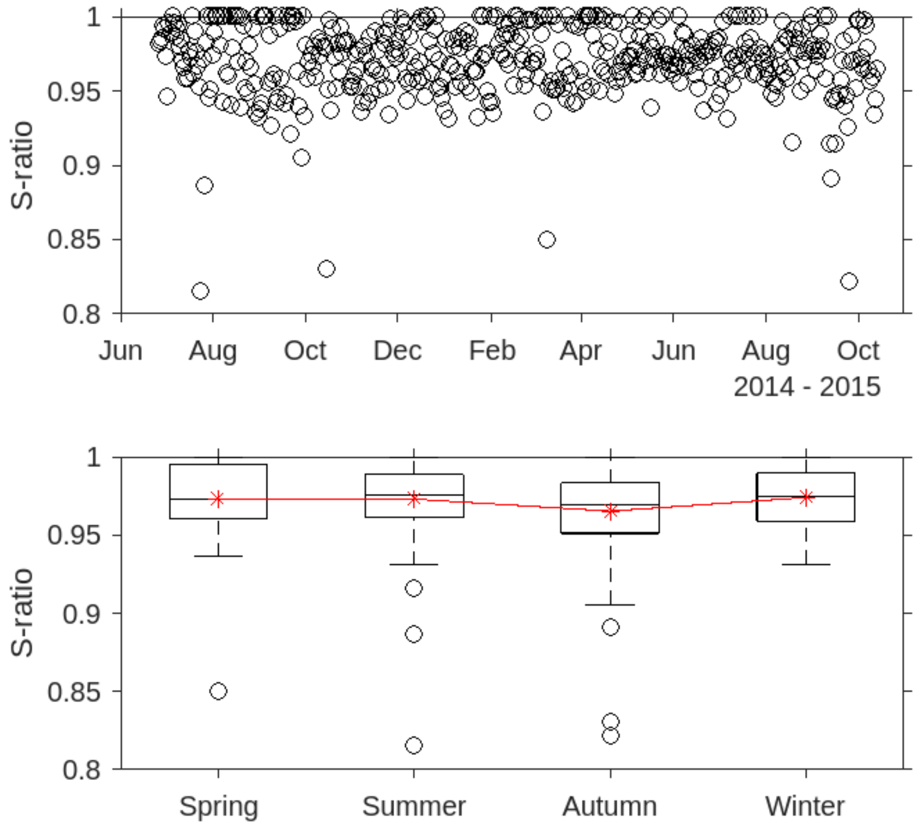

Figure S2. Time series and seasonal trend of S-ratio obtained from daily $PM_{2.5}$ samples collected by a MAAP. Spring: Mar–May; Summer: Jun–Aug; Autumn: Sep–Nov; Winter: Dec–Feb. Red marker (*) represents seasonal average (Spring: 0.973, n = 91), Summer: 0.973, n = 158), Autumn: 0.965, n = 132), Winter: 0.974, n = 90)).

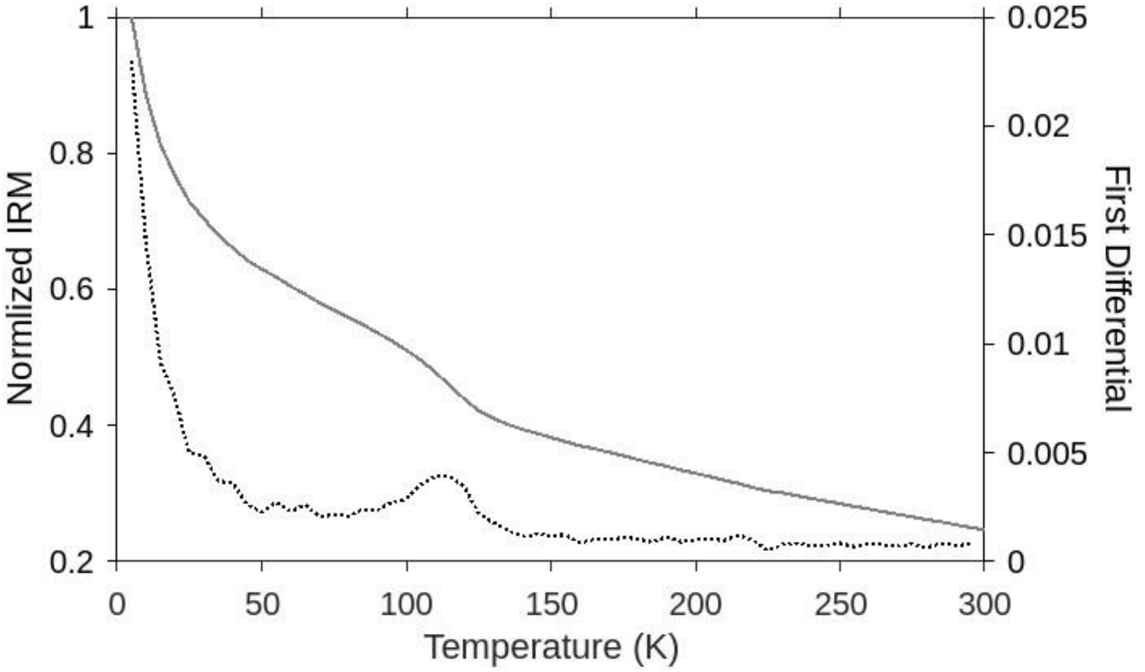

Figure S3. Temperature dependence of remanent magnetization based on low-temperature magnetic analysis for the selected HV-$PM_{2.5}$ samples. Left axis denotes remanent magnetization relative to initial remanence at 5 K, corresponding to solid line. Right axis and dashed line denote the first differential of remanence decrease line.

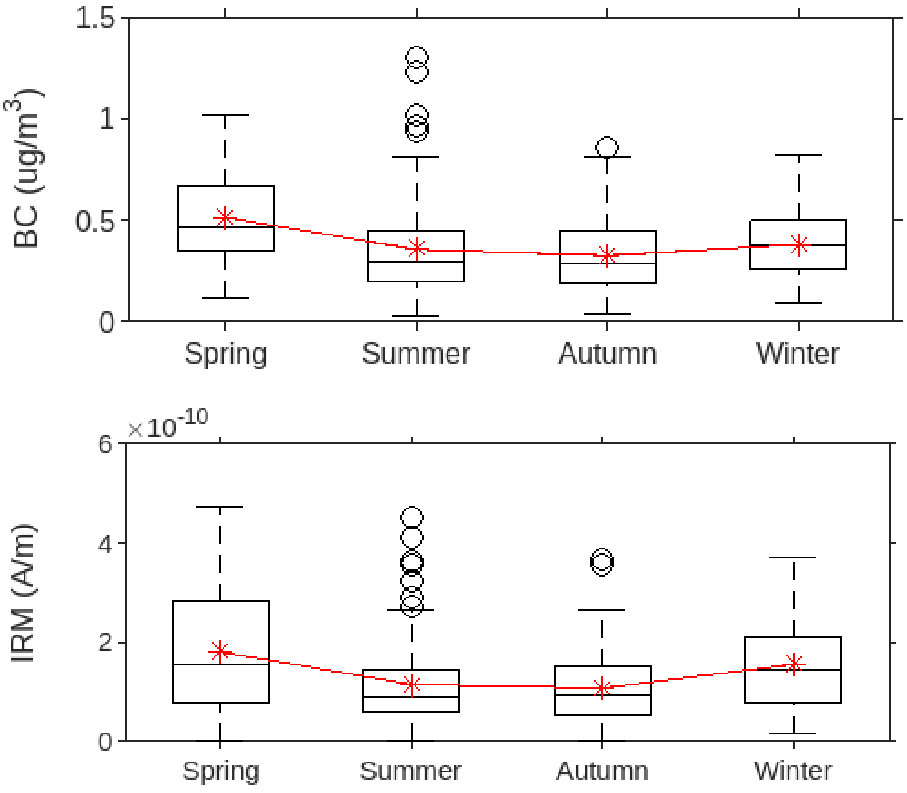

Figure S4. Seasonal trends of BC mass concentration and IRM intensity obtained from daily $PM_{2.5}$ samples collected by a MAAP (excluding points corresponding to IRM spikes). Spring: Mar–May; Summer: Jun–Aug; Autumn: Sep–Nov; Winter: Dec–Feb. Red marker (*) represents seasonal average (Spring: 0.51 μg/m^3 and 1.82 × 10^{-10} A/m (n = 69); Summer: 0.36 μg/m^3 and 1.14 × 10^{-10} A/m (n = 119); Autumn: 0.33 μg/m^3 and 1.01 × 10^{-10} A/m (n = 99); Winter: 0.38 μg/m^3 and 1.55 × 10^{-10} A/m (n = 66) for BC and IRM, respectively).

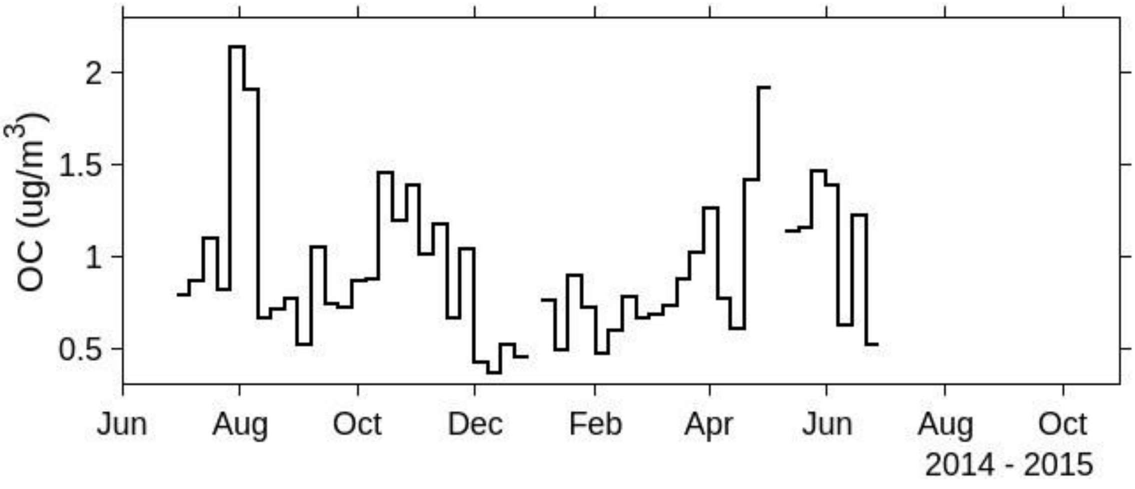

Figure S5. Temporal variation of OC mass concentration obtained from weekly HV samples.

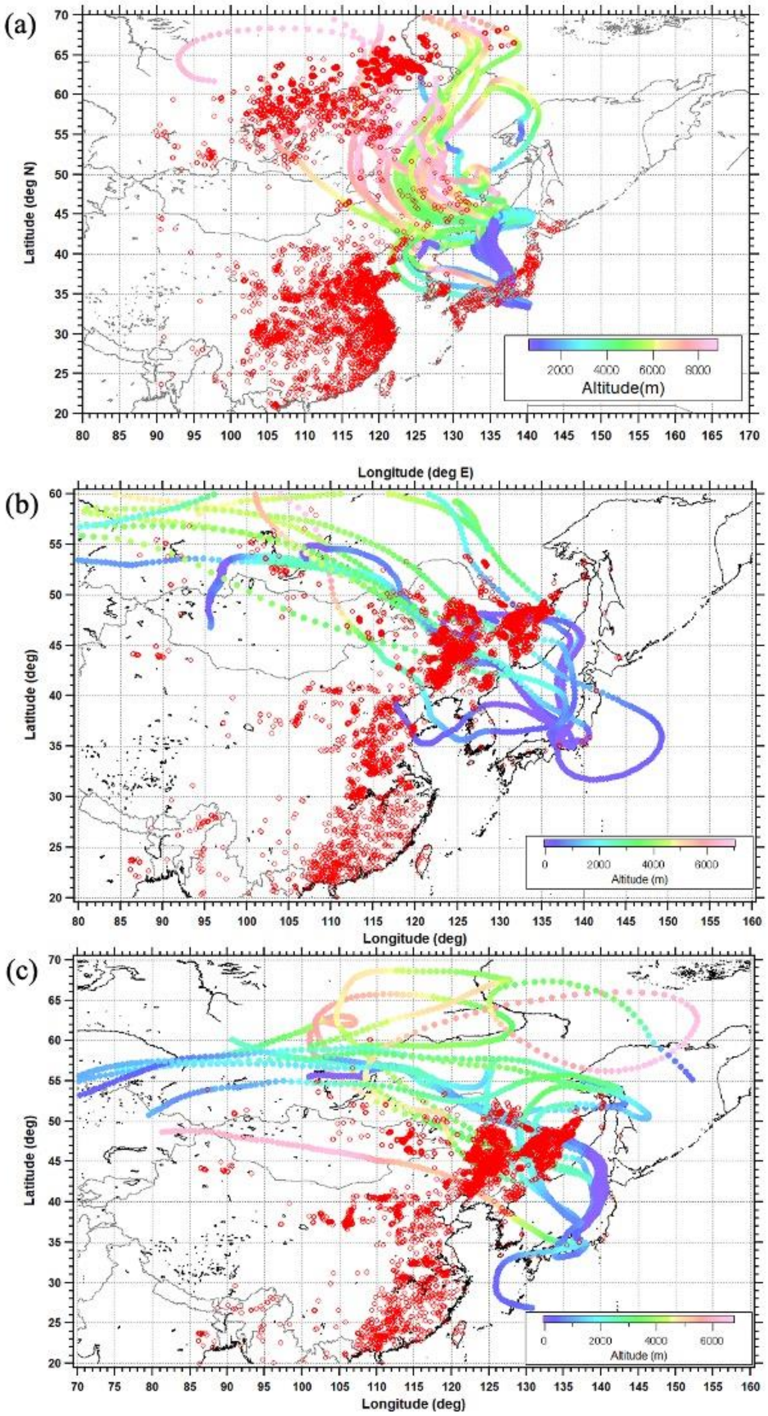

Figure S6. Backward trajectories and MODIS fire spots corresponding to the periods including the remarkable ^{14}C increase in (a) summer (July 27–30) and (b-c) autumn (October 15–22, and 22–29, respectively), 2014. Individual trajectory starts from NOTOGRO (37.45° N, 137.36° E, 500 m altitude) at 0000LST and 1200LST and goes back five days (120 h).

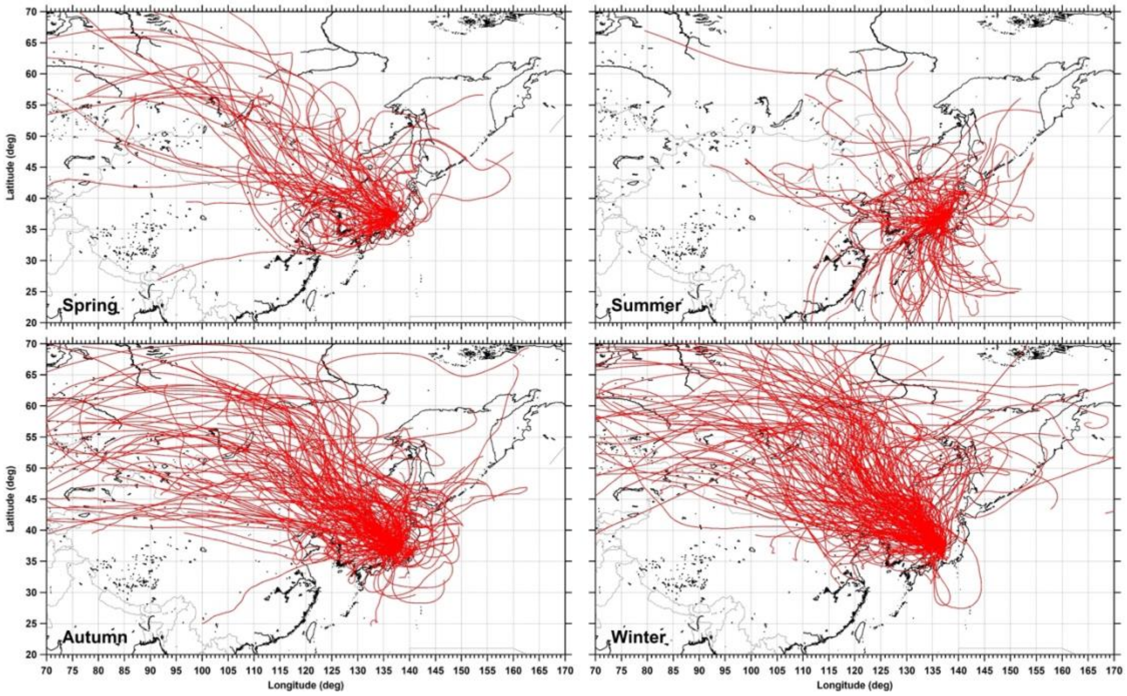

Figure S7. Daily backward trajectories for each season: Spring (Mar–May), Summer (Jun–Aug), Autumn (Sep–Nov), and Winter (Dec–Feb). Individual trajectory starts from NOTOGRO (37.45° N, 137.36° E, 500 m altitude) at 0000LST and goes back five days (120 h).

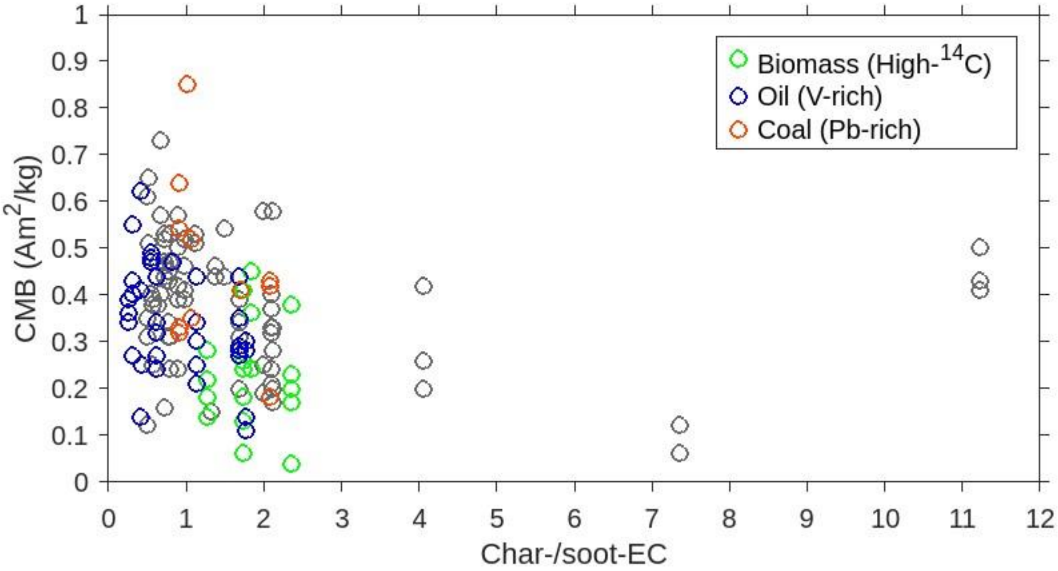

Figure S8. Relationship between char-/soot-EC and CMB. Char- and soot-EC were determined by subtracting pyrolyzed organic carbon (measured at 550˚C under a He-atmosphere) from EC1 (measured at 550˚C under an O_2/He-atmosphere), and by summing EC2 (700˚C, O_2/He) and EC3 (850˚C, O_2/He), respectively.

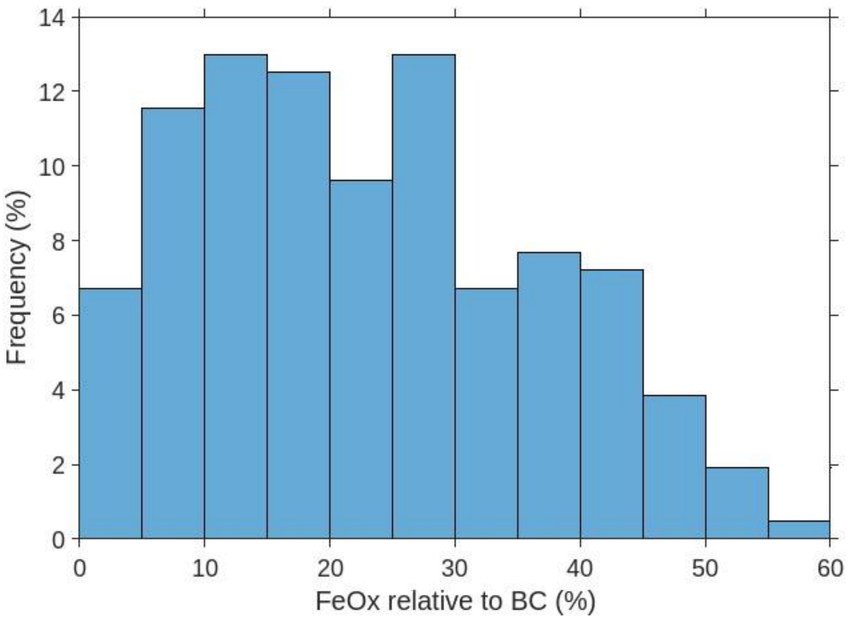

Figure S9. Frequency distribution of FeO_x relative to BC in mass concentration.

6. Supplementary Table

Table S1. The ranges (averages) of ^{14}C concentration

	N	^{14}C (pMC)
Spring<br>Mar–May	7	65.9–77.5<br>(72.1)
Summer<br>Jun–Aug	8	67.3–89.6<br>(72.8)
Autumn<br>Sep–Nov	7	71.9–89.5<br>(81.2)
Winter<br>Dec–Feb	5	57.4–71.4<br>(62.9)
Total	27	57.4–89.6<br>(73.0)

7. References

(1) Ikemori, F.; Honjyo, K.; Yamagami, M.; Nakamura, T. Influence of Contemporary Carbon Originating from the 2003 Siberian Forest Fire on Organic Carbon in PM2.5 in Nagoya, Japan. Sci. Total Environ. 2015, 530–531, 403–410. https://doi.org/10.1016/j.scitotenv.2015.05.006.

(2) Minami, M.; Kato, T.; Miyata, Y.; Nakamura, T.; Hua, Q. Small-Mass AMS Radiocarbon Analysis at Nagoya University. Nucl. Instruments Methods Phys. Res. Sect. B Beam Interact. with Mater. Atoms 2013, 294, 91–96. https://doi.org/10.1016/j.nimb.2012.02.036.

(3) Stein, A. F.; Draxler, R. R.; Rolph, G. D.; Stunder, B. J. B.; Cohen, M. D.; Ngan, F. Noaa's Hysplit Atmospheric Transport and Dispersion Modeling System. Bull. Am. Meteorol. Soc. 2015, 96 (12), 2059–2077. https://doi.org/10.1175/BAMS-D-14-00110.1.

(4) Rolph, G.; Stein, A.; Stunder, B. Real-Time Environmental Applications and Display SYstem: READY. Environ. Model. Softw. 2017, 95, 210–228. https://doi.org/10.1016/j.envsoft.2017.06.025.

(5) Tsuchiya, N.; Kato, S.; Kawasaki, K.; Nakano, T.; Kaneyasu, N.; Matsuki, A. Sources of Aeolian Magnetite at a Remote Site in Japan: Dominantly

Asian Desert Dust or Anthropogenic Emissions? Atmos. Environ. 2023, 314, 120093. https://doi.org/10.1016/j.atmosenv.2023.120093.

(6) Revuelta, M. A.; McIntosh, G.; Pey, J.; Pérez, N.; Querol, X.; Alastuey, A. Partitioning of Magnetic Particles in PM10, PM2.5 and PM1 Aerosols in the Urban Atmosphere of Barcelona (Spain). Environ. Pollut. 2014, 188, 109–117. https://doi.org/10.1016/j.envpol.2014.01.025.

(7) Kawasaki, K.; Horikawa, K.; Sakai, H. Magnetic Biomonitoring of Roadside Pollution in the Restricted Midagahara Area of Mt. Tateyama, Toyama, Japan. Environ. Sci. Pollut. Res. 2017, 24 (11), 10313–10325. https://doi.org/10.1007/s11356-017-8702-5.

(8) Verwey, E. J. W. Electronic Conduction of Magnetite (Fe3O4) and Its Transition Point at Low Temperatures. Nature. 1939, pp 327–328. https://doi.org/10.1038/144327b0.

(9) Gapeyev, A.; Tsel'movich, V. Stages of Oxidation of Titanomagnetite Grains in Igneous Rocks (in Russian). Viniti N, Moscow. 1988, 1331-B89, 3–8.

(10) Özdemir, Ö.; Dunlop, D. J.; Moskowitz, B. M. The Effect of Oxidation on the Verwey Transition in Magnetite. Geophys. Res. Lett. 1993, 20 (16),

1671–1674. https://doi.org/10.1029/93GL01483.

(11) Yang, H.; Zhang, Q.; Wu, J.; Liu, L.; Wang, D.; Lu, D.; Wang, W.; Min, K.; Zhang, W.; Liu, Q.; Yang, Y.; Jiang, G. Evolution of Magnetic Particulate Matter during Its Emission Process in Thermal Power Plants. Environ. Sci. Nano 2022, 115 (1), 1–7. https://doi.org/10.1039/D2EN00808D.

(12) Peters, C.; Dekkers, M. J. Selected Room Temperature Magnetic Parameters as a Function of Mineralogy, Concentration and Grain Size. Phys. Chem. Earth 2003, 28 (16–19), 659–667. https://doi.org/10.1016/S1474-7065(03)00120-7.

(13) Gonet, T.; Maher, B. A.; Kukutschová, J. Source Apportionment of Magnetite Particles in Roadside Airborne Particulate Matter. Sci. Total Environ. 2021, 752, 141828. https://doi.org/10.1016/j.scitotenv.2020.141828.

(14) Yang, F.; He, K.; Ye, B.; Chen, X.; Cha, L.; Cadle, S. H.; Chan, T.; Mulawa, P. A. One-Year Record of Organic and Elemental Carbon in Fine Particles in Downtown Beijing and Shanghai. Atmos. Chem. Phys. 2005, 5 (6), 1449–1457. https://doi.org/10.5194/acp-5-1449-2005.

(15) Uchida, M.; Mantoku, K.; Kumata, H.; Kaneyasu, N.; Handa, D.; Arakaki, T.; Kobayashi, T.; Hatakeyama, S.; Shibata, Y.; Kawamura, K. Source Apportionment of Black Carbon Aerosols by Isotopes (14C and 13C) and Bayesian Modeling from Two Remote Islands in East Asian Outflow Region. Nucl. Instruments Methods Phys. Res. Sect. B Beam Interact. with Mater. Atoms 2023, 538, 64–74. https://doi.org/10.1016/j.nimb.2023.02.002.

(16) Takahashi, K.; Hirabayashi, M.; Tanabe, K.; Shibata, Y.; Nishikawa, M.; Sakamoto, K. Radiocarbon Content in Urban Atmospheric Aerosols. Water. Air. Soil Pollut. 2007, 185 (1–4), 305–310. https://doi.org/10.1007/s11270-007-9454-5.

(17) Simoneit, B. R. T.; Schauer, J. J.; Nolte, C. G.; Oros, D. R.; Elias, V. O.; Fraser, M. P.; Rogge, W. F.; Cass, G. R. Levoglucosan, a Tracer for Cellulose in Biomass Burning and Atmospheric Particles. Atmos. Environ. 1999, 33 (2), 1–10.

(18) Puxbaum, H.; Caseiro, A.; Sánchez-Ochoa, A.; Kasper-Giebl, A.; Claeys, M.; Gelencsér, A.; Legrand, M.; Preunkert, S.; Pio, C. A. Levoglucosan Levels at Background Sites in Europe for Assessing the Impact of Biomass

Combustion on the European Aerosol Background. J. Geophys. Res. Atmos. 2007, 112 (23), 1–11. https://doi.org/10.1029/2006JD008114.

(19) Jung, J.; Lyu, Y.; Lee, M.; Hwang, T.; Lee, S.; Oh, S. Impact of Siberian Forest Fires on the Atmosphere over the Korean Peninsula during Summer 2014. Atmos. Chem. Phys. 2016, 16 (11), 6757–6770. https://doi.org/10.5194/acp-16-6757-2016.

(20) Engling, G.; Carrico, C. M.; Kreidenweis, S. M.; Collett, J. L.; Day, D. E.; Malm, W. C.; Lincoln, E.; Min Hao, W.; Iinuma, Y.; Herrmann, H. Determination of Levoglucosan in Biomass Combustion Aerosol by High-Performance Anion-Exchange Chromatography with Pulsed Amperometric Detection. Atmos. Environ. 2006, 40, 299–311. https://doi.org/10.1016/j.atmosenv.2005.12.069.

(21) Lewis, C. W.; Klouda, G. A.; Ellenson, W. D. Radiocarbon Measurement of the Biogenic Contribution to Summertime PM-2.5 Ambient Aerosol in Nashville, TN. Atmos. Environ. 2004, 38 (35), 6053–6061. https://doi.org/10.1016/j.atmosenv.2004.06.011.

(22) Martinelli, L. A.; Camargo, P. B.; Lara, L. B. L. S.; Victoria, R. L.; Artaxo, P. Stable Carbon and Nitrogen Isotopic Composition of Bulk Aerosol

Particles in a C4 Plant Landscape of Southeast Brazil. Atmos. Environ. 2002, 36 (14), 2427–2432. https://doi.org/10.1016/S1352-2310(01)00454-X.

(23) Moura, J. M. S.; Martens, C. S.; Moreira, M. Z.; Lima, R. L.; Sampaio, I. C. G.; Mendlovitz, H. P.; Menton, M. C. Spatial and Seasonal Variations in the Stable Carbon Isotopic Composition of Methane in Stream Sediments of Eastern Amazonia. Tellus, Ser. B Chem. Phys. Meteorol. 2008, 60B (1), 21–31. https://doi.org/10.1111/j.1600-0889.2007.00322.x.

(24) Cao, J. ji; Chow, J. C.; Tao, J.; Lee, S. cheng; Watson, J. G.; Ho, K. fai; Wang, G. hui; Zhu, C. shu; Han, Y. ming. Stable Carbon Isotopes in Aerosols from Chinese Cities: Influence of Fossil Fuels. Atmos. Environ. 2011, 45 (6), 1359–1363. https://doi.org/10.1016/j.atmosenv.2010.10.056.

(25) Widory, D. Combustibles, Fuels and Their Combustion Products: A View through Carbon Isotopes. Combust. Theory Model. 2006, 10 (5), 831–841. https://doi.org/10.1080/13647830600720264.

(26) Miyazaki, Y.; Coburn, S.; Ono, K.; Ho, D. T.; Pierce, R. B.; Kawamura, K.; Volkamer, R. Contribution of Dissolved Organic Matter to Submicron

Water-Soluble Organic Aerosols in the Marine Boundary Layer over the Eastern Equatorial Pacific. Atmos. Chem. Phys. 2016, 16 (12), 7695–7707. https://doi.org/10.5194/acp-16-7695-2016.

(27) Kim, S. W.; Saitoh, S. I.; Ishizaka, J.; Isoda, Y.; Kishino, M. Temporal and Spatial Variability of Phytoplankton Pigment Concentrations in the Japan Sea Derived from CZCS Images. Journal of Oceanography. 2000, pp 527–538. https://doi.org/10.1023/A:1011148910779.

(28) Quinn, P. K.; Bates, T. S.; Schulz, K. S.; Coffman, D. J.; Frossard, A. A.; Russell, L. M.; Keene, W. C.; Kieber, D. J. Contribution of Sea Surface Carbon Pool to Organic Matter Enrichment in Sea Spray Aerosol. Nat. Geosci. 2014, 7 (3), 228–232. https://doi.org/10.1038/ngeo2092.

(29) Li, X.; Wang, S.; Duan, L.; Hao, J.; Li, C.; Chen, Y.; Yang, L. Particulate and Trace Gas Emissions from Open Burning of Wheat Straw and Corn Stover in China. Environ. Sci. Technol. 2007, 41 (17), 6052–6058. https://doi.org/10.1021/es0705137.

(30) Huang, X.; Li, M.; Li, J.; Song, Y. A High-Resolution Emission Inventory of Crop Burning in Fields in China Based on MODIS Thermal Anomalies/Fire Products. Atmos. Environ. 2012, 50, 9–15.

https://doi.org/10.1016/j.atmosenv.2012.01.017.

(31) Sun, J.; Peng, H.; Chen, J.; Wang, X.; Wei, M.; Li, W.; Yang, L.; Zhang, Q.; Wang, W.; Mellouki, A. An Estimation of CO2 Emission via Agricultural Crop Residue Open Field Burning in China from 1996 to 2013. J. Clean. Prod. 2016, 112, 2625–2631. https://doi.org/10.1016/j.jclepro.2015.09.112.
